# Microencapsulated and Ready-to-Eat Beetroot Soup: A Stable and Attractive Formulation Enriched in Nitrate, Betalains and Minerals

**DOI:** 10.3390/foods12071497

**Published:** 2023-04-02

**Authors:** Lucileno Rodrigues da Trindade, Diego dos Santos Baião, Davi Vieira Teixeira da Silva, Cristine Couto Almeida, Fernanda Petzold Pauli, Vitor Francisco Ferreira, Carlos Adam Conte-Junior, Vania Margaret Flosi Paschoalin

**Affiliations:** 1 Laboratory of Advanced Analysis in Biochemistry and Molecular Biology (LAABBM), Department of Biochemistry, Chemistry Institute, Federal University of Rio de Janeiro (UFRJ), Avenida Athos da Silveira Ramos 149, Cidade Universitaria, Rio de Janeiro 21941-909, Brazil; 2 Graduate Studies in Food Science (PPGCAL), Institute of Chemistry (IQ), Federal University of Rio de Janeiro (UFRJ), Cidade Universitria, Rio de Janeiro 21941-909, Brazil; 3 Graduate Studies in Chemistry (PGQu), Institute of Chemistry (IQ), Federal University of Rio de Janeiro (UFRJ), Cidade Universitária, Rio de Janeiro 21941-909, Brazil; 4 Center for Food Analysis (NAL), Technological Development Support Laboratory (LADETEC), Federal University of Rio de Janeiro (UFRJ), Cidade Universitaria, Rio de Janeiro 21941-598, Brazil; 5 Institute of Chemistry (IQ), Fluminense Federal University, R. Dr. Mario Vianna, 523, Niterói 24210-141, Brazil,

**Keywords:** starch, maltodextrin, bioactive compounds, sensory attributes, technological performance, shelf life, SEM

## Abstract

Beetroot is a tuber rich in antioxidant compounds, i.e., betanin and saponins, and is one of the main sources of dietary nitrate. The aim of the present study was to microencapsulate a ready-to-eat beetroot soup by lyophilization using different encapsulating agents, which supply the required amount of bioactive nutrients. Particle size distributions ranged from 7.94 ± 1.74 to 245.66 ± 2.31 µm for beetroot soup in starch and from 30.56 ± 1.66 to 636.34 ± 2.04 µm in maltodextrin. Microparticle yields of powdered beetroot soup in starch varied from 77.68% to 88.91%, and in maltodextrin from 75.01% to 80.25%. The NO_3_^−^ and total betalain contents at a 1:2 ratio were 10.46 ± 0.22 mmol·100 g^−1^ fresh weight basis and 219.7 ± 4.92 mg·g^−1^ in starch powdered beetroot soup and 8.43 ± 0.09 mmol·100 g^−1^ fresh weight basis and 223.9 ± 4.21 mg·g^−1^ in maltodextrin powdered beetroot soup. Six distinct minerals were identified and quantified in beetroot soups, namely Na, K, Mg, Mn, Zn and P. Beetroot soup microencapsulated in starch or maltodextrin complied with microbiological quality guidelines for consumption, with good acceptance and purchase intention throughout 90 days of storage. Microencapsulated beetroot soup may, thus, comprise a novel attractive strategy to offer high contents of bioaccessible dietary nitrate and antioxidant compounds that may aid in the improvement of vascular-protective effects.

## 1. Introduction

Vegetables included in a healthy diet can protect against coronary heart diseases and reduce the risk of ischemia [1]. Vegetables are a source of fibers, vitamins, minerals and biologically active compounds. Among the several bioactive molecules available in nature, phenolic compounds, comprising secondary metabolites of roots and edible plants, and nitrate (NO_3_^−^), a negatively charged chemical compound formed by a single nitrogen atom linked to three oxygen atoms, are associated with decreased risks for chronic, degenerative and cardiovascular diseases when consumed regularly [2,3,4].

Red beetroot, *Beta vulgaris* L., a tuberous plant belonging to the *Chenopodiaceae* family, is a source of NO_3_^−^, phenolic compounds and betalains, as well as dietary fiber and minerals (potassium, sodium, iron, copper, magnesium, calcium, phosphorus and zinc) [5,6,7,8]. Betalains are heterocyclic compounds and water-soluble nitrogen pigments responsible for conferring various types of coloration not only in red beets, but also in flowers, vegetables and fruits, and have shown in vitro and in vivo antioxidant capability [7,9]. Red beetroots are also rich in mineral salts that contribute to the proper functioning of the body, as components in the formation and maintenance of bones in the human body, as cofactors or regulators of enzymatic reactions, acting in the conduction of nerve impulses, coagulation, the maintenance of osmotic balance, the transfer of substances through membranes and in the process of cellular respiration. The lack of mineral salts in the body of living beings can be fatal, as it significantly alters metabolism [10]. Furthermore, strong evidence suggests that the consumption of red beetroot, one of the most important sources of dietary NO_3_^−^, improves vascular function by converting NO_3_^−^ to NO [11,12,13,14,15,16]. After being ingested, nitrate is absorbed at the proximal portion of the small intestine, possibly the jejunum, into the bloodstream or tissues, where it accumulates along with endogenous NO_3_^−^. Dietary NO_3_^−^ increases rapidly in plasma, and about 60% of the absorbed NO_3_^−^ is excreted in the urine, while 25% is extracted by the salivary glands, concentrated in the saliva through the entero-salivary cycle. In the oral cavity, NO_3_^−^ is reduced to nitrite (NO_2_^−^) through the action of the nitrate-reductase enzyme, expressed by oral commensal bacteria that use this anion as a terminal electron acceptor to generate ATP or to incorporate it into their biomass [11,17]. Subsequently, upon reaching the gastric acid medium, NO_2_^−^ is protonated, forming nitrous acid (HNO_2_), which spontaneously decomposes (non-enzymatically) to NO and other bioactive nitrogen oxides, such as nitrogen dioxide (NO_2_), dinitrogen trioxide (N_2_O_3_) and nitrosonium ion (NO^+^) [11,15]. As dietary NO_3_^−^ can be used as a precursor for endogenous NO synthesis, new physiological, therapeutic and nutritional approaches concerning this anion have been developed. The accumulation of NO_3_^−^ and NO_2_^−^ from dietary sources takes place from these anions synthesized endogenously through the L-arginine/NO pathway [8,15,18]. Most NO_3_^−^ is lost by renal clearance and a small part is extracted by the salivary glands, concentrating in the saliva to continue the entero-salivary cycle [19]. Additionally, a small portion of plasmatic NO_3_^−^ and NO_2_^−^ may be reduced by the enzyme xanthine oxidoreductase (XOR), similar to saliva nitrate-reductase. Xanthine oxidoreductase does not depend on O_2_ to catalyze NO formation from remaining NO_3_^−^ and NO_2_^−^, so NO can be formed under hypoxia and ischemia conditions, where XOR expression and activity are increased. NO_2_^−^ can be reduced to NO by deoxyhemoglobin (deoxyHb) and deoxymyoglobin (deoxyMb), especially under low O_2_ levels [20]. Other enzymes, such as aldehyde oxidase (AO), aldehyde dehydrogenase (ALDH), carbonic anhydrase (CA) and certain compounds such as vitamin C (Vit C) and polyphenols, display the ability to reduce plasmatic NO_2_^−^ to its bioactive form, NO [1].

The food industry has developed versatile, practical or ready-to-be-consumed fruit and vegetable products in recent years with adequate sensorial attributes and, above all, providing health benefits [21]. However, it is a challenge to formulate products that, in addition to their bioactive compound richness, must be attractive in appearance, flavor and practicality while also presented in portion sizes containing pharmacological concentrations of bioactive compounds adequate for consumption. Among the current fruit- and vegetable-processing technologies, microencapsulation seems to be capable of concentrating and stabilizing functional or bioactive compounds [22]. The packaging of functional compounds into edible polysaccharides forming micrometer-sized particles functions as a physical barrier, avoiding bioactive substance degradation or interaction with the environment and releasing bioactive compounds at controlled rates under programmed physicochemical conditions [23].

Food-grade microparticles can be obtained by lyophilization, a viable and economical process that results in the crystallization of food suspensions at low temperatures [24]. Lyophilized foodstuffs can be easily transported and marketed, dispensing with refrigeration, reducing storage costs, promoting the stability of aromatic components susceptible to oxidation and heat, maintaining the overall concentration of nutrients and bioactive compounds and also comprising a practical option for consumers [25].

Herein, in natura beetroot was used to formulate a powdered and microencapsulated lyophilized soup preserving functional compounds found in the beetroot matrix, such as dietary NO_3_^−^, betacyanins, saponins and minerals. Microparticle production yields and their characteristics, i.e., size, morphology, water activity, solubility, hygroscopicity, thermogravimetric features, infra-red absorption spectroscopy and bioactive compound composition originally found in the beetroot matrix, were determined. The sensory attributes of the powdered beetroot soups and purchase intention were evaluated, as well as shelf life and microbiological safety during 90 days of storage.

## 2. Material and Methods

### 2.1. Reagents

Standard solutions and reagents were purchased from Sigma-Aldrich Chemical Co. (St. Louis, MO, USA). All solvents, including methanol (MeOH), ethanol and acetonitrile with HPLC grade were purchased from Tedia Company Inc. (Rio de Janeiro, RJ, BRA). Agar yeast, extract glucose, chloramphenicol (YGC) and agar glucose and yeast extract were purchased from Hexis Científica (São Paulo, SP, BRA). Agar Man, Rogosa and Sharpe (MRS) was purchased from Acumedia (Lansing, MI, USA); 3M PetrifilmTM Salmonella Express Sy and 3M PetrifilmTM E. coli Count Plate were purchased from 3M Health Care (São Paulo, SP, BRA). All reagents were analytical grade. Nitric acid (Vetec, Rio de Janeiro, RJ, BRA) was purified by sub-boiling followed by distillation in a quartz still (Kürner Analysentechnik, Rosenheim, BY, DEU). The multi-element standard solution Merck IV (Merck, São Paulo, SP, BRA) comprising 29 elements in nitric acid was employed to prepare standard analytical curves. Two reference materials, certified Skimmed Milk Powder ERM^®^-BD150 and Milk Powder 1549 were used to assess method accuracy (St. Louis, MO, USA). Ultra-pure water (resistivity > 18.2 MΩ cm) obtained from a Milli-Q^®^ INTEGRAL 10 system (Millipore, Burlington MA, USA) was used to prepare all reagents.

### 2.2. Preparation of Beetroot Soup Microparticles

Beetroot (*Beta vulgaris* L.) purchased from local stories in the municipality of Rio de Janeiro, RJ, Brazil, without any signs of deterioration, such as cracks, spots or wet areas, were sanitized as recommended by the Brazilian Health Regulatory Agency ANVISA (MS, Resolution RDC nº 216, of 15 September 2004), cut into cubes, baked for 60 min at 160 °C, ground in a food processor (Philips Walita-RI1836, São Paulo, SP, BRA), cooked for 20 min at 160 °C and seasoned with leek, salt and chives (Supplementary file—Table S1). After cooking, encapsulating agents were added and the material was transferred to a L101 lyophilizer (Liobras, São Paulo, SP, BRA) at −55 °C under a vacuum pressure of 55–100 µHg for 4 to 5 days.

### 2.3. Beetroot Soup Encapsulation in Starch or Maltodextrin Microparticles

Microparticles were prepared as described by Mazuco et al. [26] using two polymers, maltodextrin D20 and unmodified commercial starch. The encapsulating agents were dissolved in 100 to 200 mL of Milli-Q water by homogenization employing Ultra-Turrax T25 equipment (Ika^®^ Labotechnik, Rio de Janeiro, RJ, BRA) at 13,500 rpm. Each encapsulating agent was mixed with the beetroot soup at different ratios (1:1, 1:2 and 1:3, *w*/*w*) by stirring and homogenization employing a GOstirrer-MS-H-Pro (São Paulo, SP, BRA) at 600 rpm for 30 min followed by lyophilization (Terroni LS6000 Lyophilizer, São Paulo, SP, BRA) where sublimation of the aqueous samples was performed for 4 to 5 days until a powdery appearance was achieved. The microencapsulated beetroot soups were then ground using a mortar and pestle and the microparticles were placed in a sealed plastic container and maintained at room temperature in a cool, dry and dark place, considering the necessary headspace in order to avoid product overflow during the sealing process.

### 2.4. Morphology and Physicochemical Characterization of Beetroot Soup Microparticles

Microparticle morphology was evaluated by scanning electron microscopy (SEM), according to Maia et al. [27]. Samples were covered with a thin layer of gold using a Balzers Union FL 9496 metallizer and deposited on double-sided carbon adhesive tape fixed to the base surface of a JEOL JSM 5310 microscope (Jeol Ltd., Akishima, TKO, JPN) at 15 kV.

The size distribution of the microparticles was determined by laser diffraction employing a Mastersizer MicroPlus MAF 2000 particle size analyzer (Malvern Panalytical Ltd., Malvern, WO, UK) from 0.02 to 2000.000 μm. Samples were dispersed in water reaching an obscuration index of 10% according to three consecutive readings and the results were expressed as d (0.1), d (0.5) and d (0.9), corresponding to the maximum size, in µm, of 10%, 50% and 90% of the analyzed particles, in addition to the “span” scattering index determination [28].

Infrared absorption spectra were acquired on an Excalibur 3100 Varian FT-IR spectrophotometer (Varian Medical Systems, Inc., São Paulo, SP, BRA) set in the infrared region, in the transmittance mode, accumulating 20 scans and 2 cm^−1^ resolution. Each sample was homogenized with KBr to form pellets employed in the spectra acquisition, according to [29].

Microparticle zeta potentials (ZP) were determined using a Zetasizer LAB (Malvern Panalytical, São Paulo, SP, BRA) at 25 °C. Samples were diluted in deionized water (1%, *w*/*v*) and analyses were recorded at mV, in triplicate.

Microencapsulation yields were evaluated considering the amount of microparticles collected after lyophilization and the total amount of each sample, according to Equation (1):

(1) $$Yield\left(\%\right)=\frac{formed\;microparticles(g)\times 100}{emulsion(g)}$$

The moisture of the lyophilized beetroot soups was determined by loss of moisture and volatile substances at 105 °C according to the Association of Official Analytical Chemists [30].

The solubility of beetroot microparticles was determined by adding 1 g of each sample to 100 mL of Milli-Q water, followed by homogenization for 5 min at 15.000 rpm and centrifuging at 3000× *g* for 5 min. A 25 mL aliquot of each supernatant was then dried in an oven at 105 °C for 5 h and the solubility (%) of each sample, corresponding to the difference in weight before and after this procedure, was calculated [28].

The water activity (*a_w_*) of beetroot microparticles was measured directly at 25 °C using a Novasina LabMaster-a_w_ analyzer (AG, Neuheimstrasse, Lachen, CHE) according to Favilla et al. [31]. Water absorption indices were calculated as described by Sosulski [32].

The instrumental color of the beetroot soup microparticles was determined according to Chandran et al. [33], using an Ultra Scan Vis colorimeter (Hunter Lab, Reston, VA, USA). Calibration was performed for white prior to the sample analysis. The CIELab color space was used to determine the color components L* (black (0) to white (100)), a* (green (−) to red (+)) and b* (blue (−) to yellow (+)). L*a*b* coordinates were determined directly on the powdered microparticles, in triplicate.

### 2.5. Beetroot Soup Microparticle-Bioactive Nutrients and Minerals

The NO_3_^−^ and NO_2_^−^ contents of the beetroot soups were evaluated by high-performance liquid chromatography (HPLC), using an apparatus comprising an automatic injector and RF-10AXL fluorescence detector (Shimadzu^®^, Chiyoda, TKO, JPN), as described by Baião et al. [5].

Essential mineral concentrations (Na, K, Mg, Mn, P and Zn) were determined according to Almeida et al. [10,34] by inductively sequential coupled plasma optical emission spectrometry (ICP-OES) with a radial view on a Horiba Jobin Yvo Ultima 2 ICP-OES (Longjumeau, FRA), equipped with a cyclonic nebulization chamber, MiraMist type nebulizer (Mira Mist CE, Burgener Research Inc., Mississauga, ON, CAN) and automatic AS 421 sampler model. Instrumental ICP-OES conditions are included in the Supplementary file—Table S2. The sample analysis methods were validated by the use of certified reference materials (CRM) (Sargasso, NIES No.9) and a four-point calibration curve to assess linearity (SpecSol stock standard solution, Quimlab Química and Metrologia^®^, São Paulo, SP, BRA). The total elemental concentrations determined in the CRMs (experimental values) were compared to certified values and the recovery values were adequate for all determined elements, ranging from 83 to 106% (Supplementary file—Table S3). The respective correlation coefficients ranged from 0.97 to 1000. The LOD (detection limit) and LOQ (quantification limit) were determined for all minerals according to the Brazilian National Institute of Metrology, Quality and Technology (INMETRO), as LOD = 3 SD blank/slope of the curve and LOQ = 10 SD blank/slope of the calibration curve (Supplementary file—Table S4).

Total saponin content was determined by the vanillin-sulfuric acid assay, as described by Baião et al. [35], where absorbances at 535 nm were recorded using a Jasco V–530 UV/VIS spectrophotometer (Jasco do Brasil, Indaiatuba, SP, BRA).

### 2.6. Beetroot Soup Composition Analysis

Soup ash, protein, lipid and total dietary fiber contents were determined according to the Association of Official Analytical Chemists [30]. Total carbohydrates were estimated by deducting the sum of moisture, ash, protein, lipids and total dietary fiber contents from 100%. Calorific values (kcal) were calculated from the approximate chemical composition data.

The hygroscopicity of the beetroot soups was determined by maintaining 500 mg of powdered beetroot soup in a desiccator containing a saturated NaCl solution (UR = 75%) at room temperature for 7 days. After this period, the microparticles were weighed and hygroscopicity expressed as the percentage of absorbed mass water [36]. Thermogravimetric analyses were performed on each sample (5 mg) using a thermogravimetric Pryris 1 analyzer (Perkin-Elmer, Waltham, MA, USA) from 25 to 800 °C, with 10 °C·min^−1^ scans under an N_2_ atmosphere (30 mL·min^−1^) [27].

The fructose, glucose, sucrose and maltose contents of the beetroot microparticles were evaluated as described by Baião et al. [35] employing an HPLC LC-20AD system (Shimadzu^®^, Chiyoda, TKO, JPN) and a refractive index detector RID-10A (Waters, Milford, MA, USA) coupled to a signal integrator CBM-20A (Shimadzu^®^, Chiyoda, TKO, JPN)

### 2.7. Microparticle-Encapsulated Betalain Extraction and Quantification

Beetroot soup betalains were determined by a modification of the method described by Kusznierewicz et al. [37]. Briefly, 4 g of the powdered microencapsulated soups was dissolved in 60 mL of 30% aqueous ethanol previously acidified to pH 5.5 with formic acid (1%). The solutions were then homogenized for 30 min and centrifuged at 15,000× *g* for 30 min at 4 °C. The supernatants were then collected and an extraction step repeated three times was combined prior to betalain quantification. Sample absorbances were recorded using a UV 2800 Shimadzu UV/Vis spectrophotometer (Chiyoda, TKO, JPN) at 538 nm and 480 nm for betacyanins and betaxanthins, respectively.

Betalain contents were determined by applying Equation (2) proposed by Stintzing et al. [38]. The employed molecular weight (Mw) and molar extinction coefficients (ε) were as follows: *Mw* = 550 g·mol^−1^, e = 60,000 L·mol^−1^ cm^−1^ for betacyanin and *Mw* = 308 g·mol^−1^, e = 48,000 L·mol^−1^ cm^−1^ for betaxanthin.

(2) $$BC\left[mg\cdot g^{-1}\right]=\frac{\left(A\times DF\times Mw\times 1000\right)}{\left(\varepsilon \times L\right)}$$

where *A* comprises absorptions at 538 nm and 480 nm for betacyanins and betaxanthins, respectively, *DF* is the dilution factor and *L* the cuvette path length.

The efficiencies of betalain microencapsulation (*EE*) in starch and maltodextrin were determined individually for each pigment (total betalains, betacyanins and betaxanthins) as described by McNamee et al. [39], according to Equation (3):

(3) $$\left(EE\%\right)=\frac{BLT,\;BCN\;or\;BTX\times 100}{BLT}$$

where *BLT* comprises total betalains and BLS supernatant betalains. To calculate the *EE*%, a 1 mL aliquot of the beetroot soup extract was diluted in 10 mL of water and total betalain quantification (betacyanins + betaxanthins mg·g^−1^) was carried out. The betalain contents of the microencapsulated beetroot soups were determined by spectrophotometry at wavelengths 538 nm and 480 nm, according to Equation (2). Encapsulation efficiency was determined by the ratio between supernatant betalain content and the content retained in the core.

### 2.8. Microbial Beetroot Soup Safety throughout 90 Days

Yeast and mold counts were performed according to the American Public Health Association (APHA) [40]. *Salmonella* spp. and *Escherichia coli* counts were evaluated by the 3 M Petrifilm^TM^ *Salmonella* Express Sy and 3 M Petrifilm^TM^ *E. coli* Count Plate (3 M Health Care) assays, respectively. Total and fecal coliforms were evaluated by the most probable number per gram (MNP·g^−1^) standard methodology. *Bacillus cereus* were determined in selective MYP medium (Plast Labor^®^, Rio de Janeiro, RJ, BRA), according to APHA [40].

### 2.9. Sensory Analysis and Purchase Intention after Beetroot Soup Production and throughout 90 Days

Non-trained judges were recruited through invitation signals posted at the Federal University of Rio de Janeiro (UFRJ) and invitations to individuals of both genders aged 18–60 years old. Before the beginning of the sensory test, each panelist signed a Free and Informed Consent Form (Institutional Ethics Committee of the Clementino Fraga Filho University Hospital, Rio de Janeiro, No. 559 15510313.5.0000.5257).

All participants received instructions for carrying out the tasting and questionnaire answering, accompanied by a researcher who remained throughout the entire procedure to clarify any doubts. The participants evaluated the features of the powdered beetroot soup within 90 days of manufacturing by considering characteristic beetroot color, texture and aroma maintenance and regular powder appearance. Each volunteer evaluated the three types of powdered beetroot soup differentiated by acronyms just after manufacturing (time 0) and after 30, 60 and 90 days of storage. Sensory evaluations were carried out in a closed cabin with white lighting and the samples were randomly served accompanied by an evaluation form. Beetroot soup formulations were evaluated individually for acceptability by assessing powder attributes (color, texture and aroma) and overall impression through a 9-point structured hedonic scale. To encourage non-trained judges to perform a more conscientious purchase intention analysis, purchase intention was scored using a 5-point hedonic scale, ranging from “I would certainly buy it” (score 5) or “I would certainly not buy it” (score 1) [41].

### 2.10. Statistical Analyses

A one-way analysis of variance (ANOVA) with repeated measures was performed to identify lyophilized combination differences employed for characterization and stability analyses. When the F value was significant, an additional post hoc test was performed by applying a Bonferroni correction analysis. Data were expressed as means ± standard deviations (SD). All statistical analyses were performed using Graphpad Prism software v. 5 for Windows^®^ (GraphPad Software, San Diego, CA, USA).

## 3. Results

### 3.1. Particle Size Distribution

The particle size distribution and the span scattering index results indicated particle diameters ranging from 7.94 ± 1.74 to 245.66 ± 2.31 µm for beetroot soup microencapsulated in starch, and from 30.56 ± 1.66 to 636.34 ± 2.04 µm in maltodextrin (Table 1). The beetroot soup microencapsulated in starch at a 1:2 ratio displayed the best particle size distribution, while the best ratio for beetroot soup microencapsulated in maltodextrin was 1:3. Entrapment at 1:2 (*w*/*w*) and 1:3 (*w*/*w*) ratios resulted in smaller-sized microparticles and a discrete span, while a 1:1 (*w*/*w*) ratio between microencapsulated beetroot soup in starch or maltodextrin favored microparticle size heterogeneity.

### 3.2. Morphology

**Figure 1:**
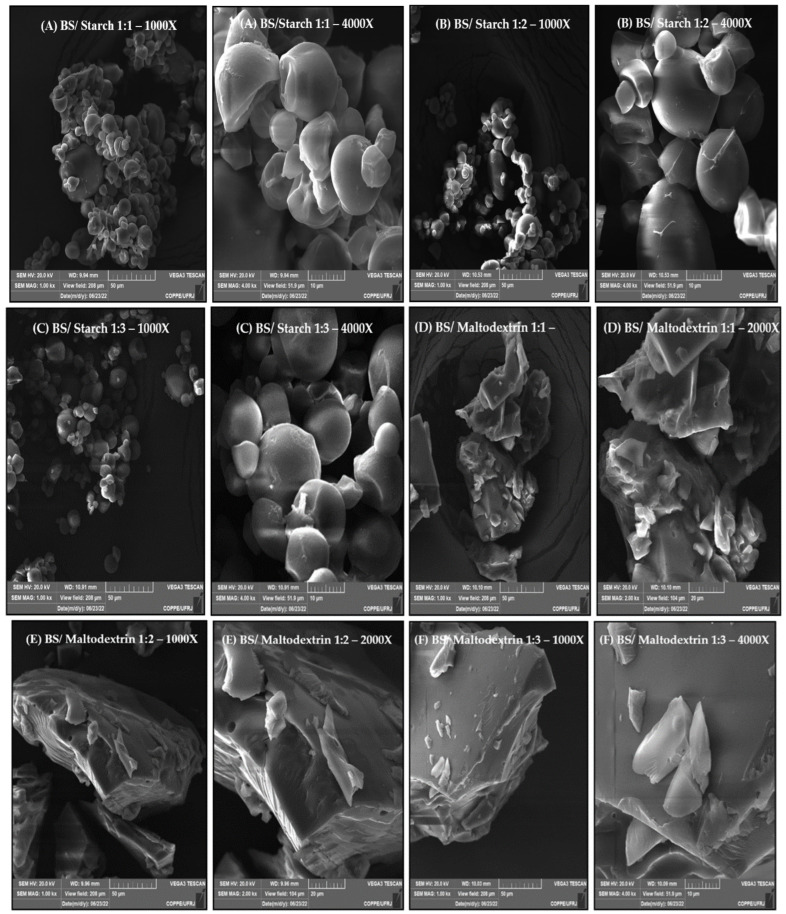

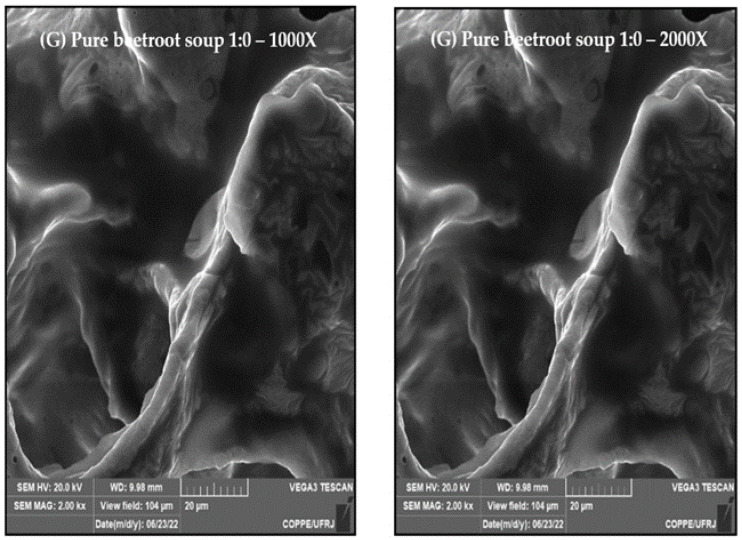
Beetroot soup microparticle micrographs following lyophilization in different encapsulating agents, 1000× and 4000× magnifications, respectively. (**A**–**C**) beetroot soup microencapsulated in starch at 1:1, 1:2 or 1:3 (*w*/*w*) ratios, respectively; (**D**–**F**) beetroot soup microencapsulated in maltodextrin at 1:1, 1:2 or 1:3 (*w*/*w*) ratios, respectively; (**G**) pure beetroot soup.

Figure 1 displays representative SEM images of microparticles encapsulated in starch and maltodextrin at 1:1, 1:2 and 1:3 (*w*/*w*) wall/core ratios. The obtained SEM images demonstrated that microparticles in starch presented spherical and irregular geometric conformations, without invaginations or roughness (Figure 1; starch 1:1, starch 1:2 and starch 1:3). Microparticles in maltodextrin presented a vitreous appearance, also with no invaginations or roughness (Figure 1; malto 1:1, malto 1:2 and malto 1:3). Pure beetroot soup SEM images, with no encapsulating matrix, differed from the others, as expected (Figure 1, pure beetroot soup).

### 3.3. Fourier-Transform Infrared Spectroscopy (FTIR)

**Figure 2:**
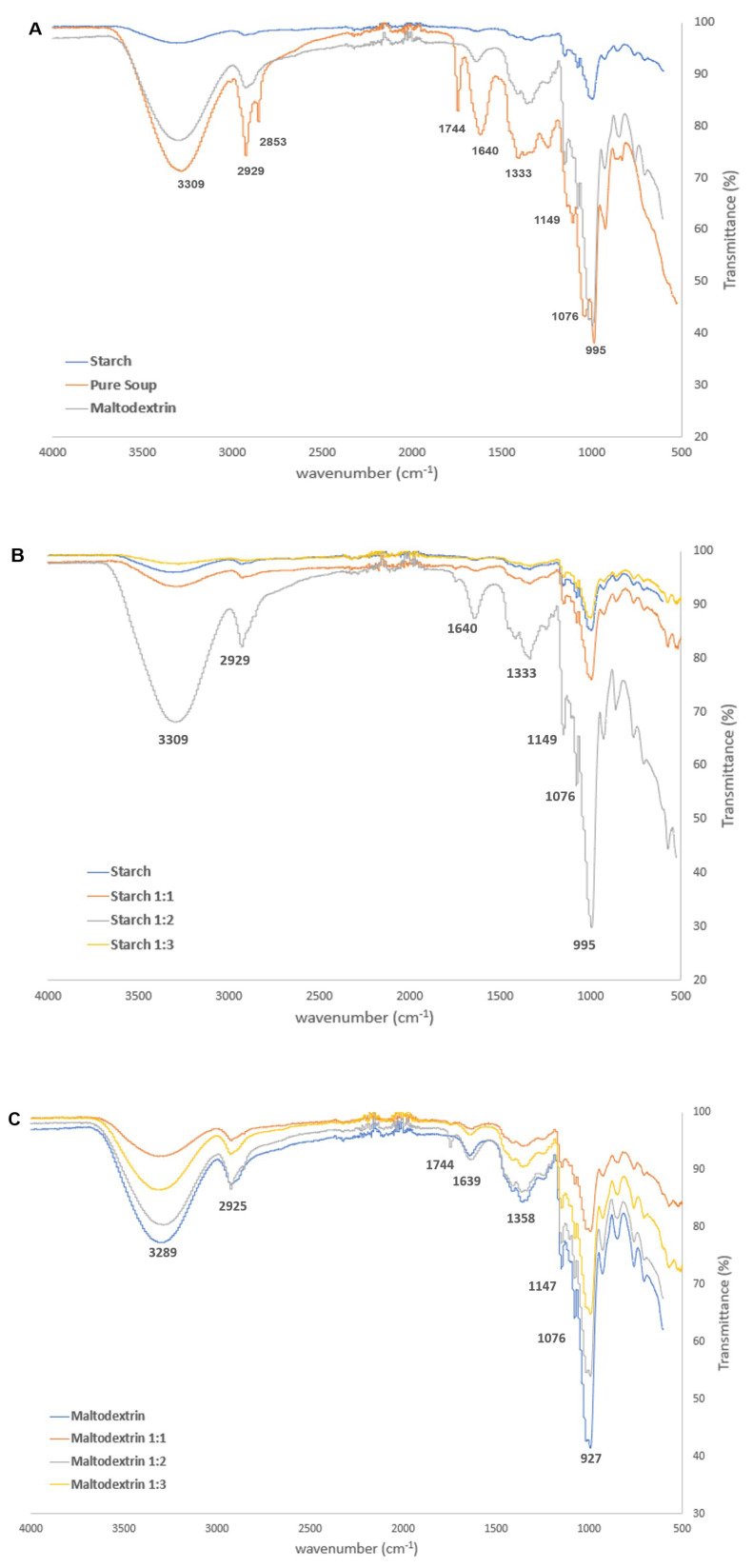
Infrared absorption spectra (FTIR) of lyophilized (powdered) beetroot soups: (**A**) beetroot soup microencapsulated in starch at 1:1, 1:2 and 1:3 ratios, (**B**) beetroot soup microencapsulated in maltodextrin at 1:1, 1:2 and 1:3 ratios and (**C**) pure beetroot soup.

The infrared absorption spectra acquired to follow chemical microparticle composition and eventual changes during the drying process (Figure 2) indicated characteristic O-H bands at 3283 cm^−1^ and aliphatic C-H groups at 2923 cm^−1^ and 2854 cm^−1^ in the beetroot matrix. Bands at 1744 cm^−1^ and 1628 cm^−1^ may be due to the C=O bond stretching of carboxylic acids and C=N imine bonds, comprising functional groups found in betanin. The bands present at 1039 cm^−1^ and 985 cm^−1^ are characteristic of the angular deformation of C-O bonds (Figure 2A).

The infrared maltodextrin spectra presented characteristic bands of maltodextrin oligomers, with the band at 3289 cm^−1^ referring to O-H bond stretching and the band at 2925 cm^−1^ indicating vibrational stretching of the C-H bonds of alkyl groups. Furthermore, the characteristic bands at 1639, 1358 and 992 cm^−1^ can be attributed to the angular straining of OH and C-H bonds and the stretching of C-O bonds, respectively (Figure 2B). The infrared starch spectra presented characteristic polysaccharide monomer bands, where the middle band at 3310 cm^−1^ results from O-H bond stretching and the band at 2929 cm^−1^ is associated to the vibrational stretching of the C-H bond from saturated carbon (C sp^3^). Furthermore, the characteristic bands present at 1333 cm^−1^ and 995 cm^−1^ were attributed to the angular deformation of C-H bonds and the stretching of C-O bonds, respectively (Figure 2C).

The spectra obtained for beetroot soup microencapsulated in maltodextrin presented bands previously observed in the single maltodextrin and beetroot soup spectra. The 1745 cm^−1^ band characteristic of the carbonyl bond present in betanin was also detected (Figure 2B). Furthermore, beetroot soup microencapsulated in starch presented bands previously observed in the polysaccharide alone, as well as a band at 1640 cm^−1^ characteristic of the amino bond present in betanin, thus confirming beetroot soup entrapment within starch microparticles (Figure 2C).

### 3.4. Characterization of Beetroot Soup Microparticles

The yield, moisture, *a_w_*, solubility and water absorption indices of microparticles produced from beetroot soup feed solutions at distinct encapsulating matrix ratios are depicted in Table 2. Powdered beetroot soup microencapsulation yields ranged from 77.68% to 88.91% using starch as the microencapsulating agent, and from 75.01% to 80.25% when maltodextrin was used. These microencapsulation yields are satisfactory, since increased yields with increasing encapsulating agent concentrations were noted, as well as solid content following the drying process. The microparticle yields were superior in starch compared to maltodextrin, probably due to the molecular structure of starch, allowing for faster drying.

The moisture of beetroot soup microparticles ranged from 3.11 ± 0.43 to 3.19 ± 0.55% in starch, and from 4.61 ± 0.95 to 4.77 ± 0.75% in maltodextrin. The microparticle moisture in starch was lower than in maltodextrin and when no encapsulating agent was added, indicating a possible technological improvement in the shelf life and overall stability of the beetroot soup product (Table 2). Furthermore, beetroot soup microparticles presented low *a_w_*, ≤0.55 for beetroot soups without encapsulant agents and ≤0.15 and ≤0.50 microencapsulated in starch and maltodextrin, respectively, with the exception of the 1:3 (*w*/*w*) ratio in maltodextrin, which reached <0.69 (Table 2). The water solubilities of beetroot soup microencapsulated in starch ranged from 31.77 ± 2.39% to 34.88 ± 1.55% and from 48.29 ± 2.72% to 52.35 ± 2.38% in maltodextrin. Starch microparticles presented lower solubility when compared with maltodextrin microparticles, but both presented low solubility in water, as expected for a food or pasty product such as the beetroot soup formulated herein (Table 2).

Water absorption indices ranged from 119.53 ± 1.79% to 222.17 ± 1.85% in beetroot soup microencapsulated in starch, and from 508.04 ± 1.91% to 509.83 ± 1.61% in maltodextrin microcapsules, demonstrating a higher water absorption index in maltodextrin with a significant difference when compared with starch and pure beetroot soups (Table 2).

### 3.5. Zeta Potential

Pure beetroot soup exhibited a low surface charge (−8.36 ± 0.01 mV), while starch microencapsulation increased the zeta potential, reaching −21.29 ± 0.14 at 1:3 (*w*/*w*) and -20.28 ± 1.04 at the 1:2 (*w*/*w*) ratio. Furthermore, the zeta potential increased with increasing starch-to-beetroot ratios. On the other hand, encapsulation in maltodextrin promoted an increased surface charge of the soup at a magnitude greater than in starch at the 1:2 (*w*/*w*) (beetroot soup:maltodextrin) ratio (Table 2).

### 3.6. Instrumental Color Analyses

The color of the beetroot soup microparticles in starch or maltodextrin at 1:1, 1:2 and 1:3 (*w*/*w*) beetroot soup/encapsulating agent ratios and that of pure beetroot soup are presented in Table 3. According to the CIE system scale for the L* (brightness) parameter, pure beetroot soup microparticles displayed less luminosity (lower L* values) compared to beetroot soup microencapsulated in maltodextrin or starch. Both encapsulating agents conferred luminosity, with higher encapsulating agent ratios resulting in higher luminosity values. Considering b*, which evaluates hue variations from blue (−) to yellow (+), the beetroot soup microparticles presented positive values, suggesting a tendency towards yellow, with a significant difference detected when comparing pure beetroot soup and beetroot soup in starch. Furthermore, when assessing hue variations from green (−) to red (+), a* microparticle values were positive, suggesting a tendency towards red. However, the higher the encapsulating agent’s concentrations, the lower the red coloration, as expected. Beetroot soup microparticles in starch were less red when compared to the same maltodextrin ratio of pure beetroot soup.

### 3.7. NO_3_^−^, NO_2_^−^, Saponin and Mineral Contents

As expected, bioactive compound and mineral contents were reduced according to the polysaccharide amount used to microencapsulate the beetroot soups. The NO_3_^−^ contents of beetroot soup microencapsulated in starch at 1:1 and 1:2 (*w*/*w*) ratios, of 16.67 ± 0.19 and 10.46 ± 0.22 mmol·100 g^−1^ fresh weight basis, respectively, were higher than values observed for beetroot soup microencapsulated in maltodextrin (15.98 ± 0.33 and 8.43 ± 0.09 mmol·100 g^−1^ fresh weight basis). No changes in NO_2_^−^ contents were observed when comparing beetroot soup microencapsulated in starch or maltodextrin (Table 3).

The saponin content of beetroot soup microencapsulated in starch and maltodextrin decreased according to increasing encapsulating agent ratios (ratio 1:1 > ratio 1:2 > ratio 1:3). No changes were observed in saponin content when comparing beetroot soup microencapsulated in starch or maltodextrin considering the 1:1, 1:2 and 1:3 (*w*/*w*) ratios. However, pure beetroot soup (25.10 ± 0.08 and 0.76 ± 0.11 mmol·100 g^−1^ fresh weight basis, and 9921.79 ± 25.14 mg∙100 g^−1^) contained the highest NO_3_^−^, NO_2_^−^ and saponin contents compared to beetroot soup microencapsulated in either starch or maltodextrin (Table 3).

Six distinct minerals were detected and quantified in beetroot cereal soups, namely Na, K, Mg, Mn, Zn and P, although the average contents of minerals in pure beetroot soup and in microencapsulated beetroot soups were highly variable for most of the detected minerals (Table 3), with Na, K and Mg as the most abundant. The mineral contents in pure beetroot soup reached 1779.46 ± 7.18 mg∙100 g^−1^, superior to those detected in beetroot soup microencapsulated in both starch and maltodextrin.

### 3.8. Final Product Characterization

The best beetroot soup microparticle formulations with and without encapsulating agents were chosen for detailed characterizations for proximate composition, betalain microencapsulation efficiency and a microbiological stability test and sensory analysis assay based on previously obtained results. Yield, moisture, *a_w_*, solubility, water absorption index and particle size distribution were not considered parameters of choice, as all microparticles exhibited similar and desirable values. Based on these criteria, formulations microencapsulated with starch and maltodextrin at the 1:2 ratio formulations were selected due to the morphology, zeta potential and rheological characteristics. 

**Figure 3:**
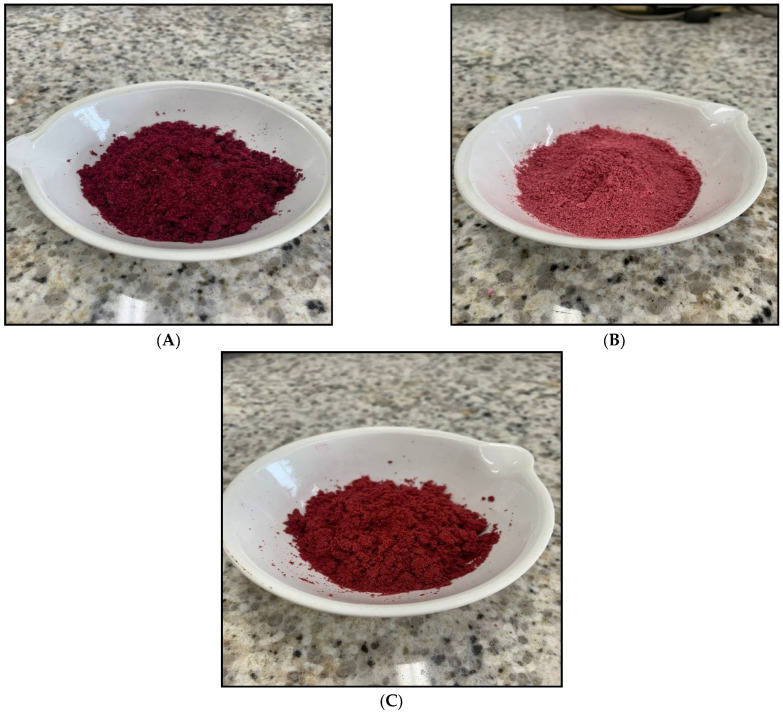
Lyophilized beetroot soup containing no microencapsulating agent (**A**) in starch (**B**) and in maltodextrin (**C**) microencapsulating agents at a 1:2 ratio (*w*/*w*).

Beetroot soup lyophilized with no encapsulating agent and beetroot soup microencapsulated with starch and maltodextrin at the 1:2 (*w*/*w*) ratio comprised a solid, granular and heterogeneous powder. Beetroot soup lyophilization with no encapsulating agent was dark red, while beetroot soup microencapsulated with starch at the 1:2 (*w*/*w*) ratio was clear red and beetroot soup microencapsulated with maltodextrin at the 1:2 (*w*/*w*) ratio was bright red. Powdered beetroot soups in the presence of encapsulating agents exhibit a sweeter and slightly earthy odor and aroma when compared to the product prepared without any encapsulating agent. The texture of the powdered soups encapsulated with starch and maltodextrin is solid, regular and slightly rough, whereas the texture of powdered soups with no encapsulating agent is solid, irregular and very rough (Figure 3).

### 3.9. Centesimal Composition and Sugar Contents

Beetroot soup microencapsulated in starch presented the lowest moisture content, 3.15 ± 0.81%, compared with pure beetroot soup and beetroot soup microencapsulated in maltodextrin, both presenting very similar moisture contents of 4.50 ± 0.34% and 4.65 ± 0.53%, respectively. The lipid contents of beetroot soup microencapsulated in starch and maltodextrin, 2.27 ± 0.82 and 2.19 ± 0.75 g·100 g^−1^, respectively, were 2-fold lower than pure beetroot soup, as expected, reaching 5.41 ± 1.11 g·100 g^−1^, while the total energy and carbohydrate contents of both microencapsulated beetroot soups were higher than pure beetroot soup. No variations in ashes, energy, carbohydrate, protein, lipid, total dietary fibers, total sugars, fructose, glucose and sucrose contents of beetroot soup microencapsulated in either starch or maltodextrin were observed. Furthermore, beetroot soup microencapsulated in starch and maltodextrin presented ash contents of 2.22 ± 0.44 and 1.83 ± 0.37% and total dietary fibers of 3.57 ± 0.37 and 3.40 ± 0.36 g·100 g^−1^, respectively (Supplementary file—Table S5).

### 3.10. Total Betalain, Betacyanin and Betaxanthin Encapsulation Efficiencies

The total betalain, betacyanin and betaxanthin encapsulation efficiencies of beetroot soup encapsulated at the 1:2 (*w*/*w*) ratio are depicted in Table 4. Pure beetroot soup contained higher betacyanin (258.1 ± 0.45 mg·g^−1^), betaxanthin (142.2 ± 2.63 mg·g^−1^) and total betalain (400.2 ± 2.17 mg·g^−1^) contents compared to beetroot soup microencapsulated in starch and maltodextrin (1:2). The encapsulation efficiencies were higher than 50% for betacyanins, betaxanthins and total betalains considering beetroot soup encapsulated in both starch and maltodextrin. Furthermore, no significant differences were observed in betacyanin, betaxanthin and total betalain contents and, consequently, in the encapsulation efficiency of these compounds among the microencapsulated beetroot soups.

### 3.11. Microbiological Analyses (Shelf Life)

The shelf lives of pure beetroot soup and beetroot soup microencapsulated in starch and maltodextrin at a 1:2 (*w*/*w*) ratio were evaluated by microbial quality testing for 90 days (Supplementary file—Table S6). No yeast, mold, *Salmonella* spp., *E. coli*, *Bacillus cereus*, total coliform or thermotolerant coliform contaminations were detected in the first 60 days of processing. These findings are under the maximum safe limits established by the current Brazilian sanitary legislation RDC resolution N°. 331 for powdered food for human consumption [42]. Although mold and yeast analyses are not required by the current Brazilian legislation, their presence was also assessed due to their potential for powdered product spoilage. Mold and yeast were detected in pure beetroot soup after 60 and 90 days of manufacturing, at 2.7 × 10^3^ CFU·g^−1^ and 4.5 × 10^3^ CFU·g^−1^, respectively. Mold and yeast were detected at 1.1 × 10^2^ and 1.4 × 10^2^ CFU·g^−1^, respectively, in microencapsulated beetroot soup after 90 days of manufacturing.

### 3.12. Sensory Analysis

Powdered beetroot soups were evaluated with high mean scores on all sensory attributes (Table 5). Pure beetroot soup exhibited significant color, aroma and overall acceptability differences when compared with microencapsulated beetroot soup in starch or maltodextrin 0, 30, 60 and 90 days after manufacturing. Differences in texture and purchase intention were also noted when comparing pure beetroot soup with microencapsulated beetroot soup in starch or maltodextrin 60 and 90 days after manufacturing. Color and texture attributes and the overall acceptability of the microencapsulated beetroot soups received average scores corresponding to “I really liked it” (score ≥ 8.0), indicating high product acceptance. In addition, the two microencapsulated formulations received average scores corresponding to “I liked it moderately” (score ≥ 7.0) for aroma, also indicating a high acceptance. In addition, the two microencapsulated beetroot soups received the highest purchase intent scores, corresponding to “I would probably buy it” (score ≥ 4.0) 0, 30, 60 and 90 days after manufacturing. Beetroot soups microencapsulated in starch or maltodextrin presented good scores regarding color, texture, overall acceptability and purchase intention, with no significant difference between them.

## 4. Discussion

The microencapsulated beetroot soups produced herein were carefully designed to fulfill the nutritional characteristics of a food product to be used as a long-term dietary NO_3_^−^ supplement, a natural NO precursor, as well as a source of other bioactive compounds that could act as promising adjuvants against vascular events in individuals presenting risk factors for developing CVD or those with established CVD.

Beetroot soup encapsulated in maltodextrin formed larger and more heterogenous microparticles compared to beetroot soups encapsulated in starch, as maltodextrin favors the rapid formation of a glassy surface, which allows for air expansion inside the microparticles, resulting in increased average sizes. Similar results were reported by Rezende et al. [43] concerning the encapsulation of bioactive compounds from acerola pulp by lyophilization and spray drying, with microparticle diameters ranging between 18.75–464 µm and 99.26 µm, respectively. Herein, the microparticles obtained by starch encapsulation, especially at the 1:2 ratio (*w*/*w*), were more homogeneous, with a narrower size distribution and less size variability. These results are similar to those reported by Murali et al. [44], where microparticles varying from 9.44 to 21.20 µm were obtained by the lyophilization of carrot juice using tapioca starch as an encapsulating agent.

It is important to note that microparticle size influences several product features, such as solubility, dispersibility and the release of matrix-entrapped compounds [45]. Homogeneous microparticle sizes improve the reconstitution of the lyophilized product. The best results were obtained using starch as an encapsulating agent at 1:2 and 1:3 (*w*/*w*) ratios, leading to smaller microparticles with smaller spans. The 1:1 (*w*/*w*) ratio between microencapsulated beetroot soup in starch or maltodextrin, however, favored span increase.

Microparticle microstructure, size, polydispersity and morphology during lyophilization can undergo changes depending on the encapsulating matrix composition and properties, the core:wall material ratio and drying and storage conditions [46]. No morphological differences were observed between beetroot soup microparticles prepared at different ratios in starch or maltodextrin. The polysaccharide agents, however, modified microparticle morphology, with starch microcapsules presenting a spherical and irregular geometry with few surface depressions and aggregation formed by the adhesion of smaller particles to the surface of larger ones, in contrast to the maltodextrin microparticles, which presented a vitreous appearance, as described elsewhere [47,48,49]. Furthermore, no cracks or open pores were observed in either type of microparticle. This morphology is similar to that reported when carrot juice was encapsulated by lyophilization and spray drying into wall materials such as tapioca starch, maltodextrin or gum Arabic [44].

The infrared spectra of powdered beetroot soups microencapsulated at three different starch ratios mostly displayed similar bands regardless of starch ratio and bands referring to beetroot soup, indicating that chemical component structures were preserved during the microencapsulation/lyophilization process. The band at 1640 cm^−1^, characteristic of the betanin imine bond (C=N), and the band detected at 3300 cm^−1^, indicating O-H bond stretching, also present in betanin, indicate that the betanin structure was preserved in beetroot soup microparticles [50,51]. The infrared spectra for beetroot soup microencapsulated in starch exhibited characteristic bands of the polysaccharide constituents, i.e., a medium intensity band at 3300 cm^−1^, referring to O-H bond stretching, and a band at 2929 cm^−1^, referring to the vibrational stretching of the C-H bond of saturated carbon (C sp^3^). In addition, a 1333 cm^−1^ band was also noted, which can be attributed to the angular deformation of C-H bonds. In the typical polysaccharide region, bands at 1147 cm^−1^ and 1076 cm^−1^ can be attributed to vibrational C-O bond stretching, while a band at 995 cm^−1^ is attributed to the absorption of the C-O-C bond [52,53].

Infrared maltodextrin spectra indicated a band at 3289 cm^−1^ referring to O-H bond stretching, while a band at 2925 cm^−1^ refers to the vibrational stretching of the C-H bond of an alkyl group. Furthermore, the characteristic bands at 1639 cm^−1^ and 1358 cm^−1^ can be attributed to the angular straining of OH bonds (referring to absorbed water) and angular straining of C-H bonds, respectively. Bands at 1147 cm^−1^, 1076 cm^−1^ and 927 cm^−1^ were observed in the characteristic carbohydrate absorption region, referring to C-O-C and C-O deformations [54,55]. The microparticle spectra of beetroot soup microencapsulated in maltodextrin, regardless of the polysaccharide ratio used for microencapsulation, can be attributed predominantly to maltodextrin. At the 1:2 (*w*/*w*) ratio, a band at 1744 cm^−1^, characteristic of pure beetroot soup, is still observed (Figure 2A), attributed to C=O bond stretching referring to the carboxylic acid groups of betanin, the main constituent of pure beetroot soup.

Microencapsulation yields increased with higher encapsulating agent concentrations, as well as higher solid contents, indicating a successful drying process. These results are satisfactory for technological improvements, particularly at the 1:2 (*w*/*w*) ratio for beetroot soup and polysaccharides. However, starch microencapsulation yields were higher than maltodextrin, maybe due to the molecular structure of starch, which allows for fast sample drying. Indeed, lower final product drying times may be considered an advantage concerning the beetroot soup lyophilized process [28].

The *a_w_* of beetroot soups microencapsulated in maltodextrin, and mainly in starch, do not favor the growth of pathogenic and deteriorating microorganisms such as bacteria, yeast and fungi, as these microorganisms proliferate at *a_w_* > 0.85%, 0.80% and 0.62%, respectively. Pena et al. [56] evaluated the hygroscopic behavior of powdered *açaí,* the fruit from the *Euterpe oleracea* palm, prepared by spray drying, and reported accelerated deterioration rates at *a_w_* ≥ 0.6%. Although chemical and enzymatic reactions can occur at *a_w_* = 0.2%, as a certain amount of water adsorbed on the powder surface in this condition allows for water to act as a solvent, this condition was not reached in starch microencapsulation at all, although the 1:3 ratio (*w*/*w*) for maltodextrin microencapsulation should be avoided. Microencapsulated beetroot soup displayed better performance than pure beetroot soup and should, therefore, be considered a safer and more stable food product.

Furthermore, the high moisture in powdered food favors deterioration by altering sensory attributes, also affecting overall product acceptability [57]. The microencapsulated beetroot soups were under the 15% threshold considered safe according to the current Brazilian sanitary legislation for powdered food [58].

The solubility of lyophilized products is an important marketing parameter and must be considered when choosing the encapsulating agent, with carbohydrates recommended to obtain high solubilization-capacity microparticles when reconstituted in water [59,60,61]. In the present study, a discrete increment in the solubility of powdered beetroot soups following maltodextrin or starch microencapsulation was observed, and the better performance of beetroot soups when reconstituted in mild-temperature water was input to these polysaccharides. It is important to note that the ability of dissolved powders to remain as a homogeneous solution in water associates to other sensorial and organoleptic attributes and can affect marketed food acceptability. 

The magnitude of the zeta potential measures colloidal system stability, as electrostatic repulsions decrease Van der Waals forces of attraction, favoring dispersion stability [62]. Colloidal dispersions displaying zeta potential > 30 mV, positive or negative, are physically stable, while microparticles with lower zeta potential tend to agglomerate [63]. The encapsulating starch and maltodextrin agents applied herein provided beetroot soup stability by increasing the negative surface charges of the beetroot soup zeta potential 2-fold. Negative charges are common to starch and maltodextrin, with negative zeta potentials reported for both catechin-starch and pequi (*Caryocar brasiliense*) oil-maltodextrin microparticles in other studies [64,65,66]. These findings demonstrate the effectiveness of microencapsulation employing starch and maltodextrin to improve beetroot soup stability.

In the present study, a color analysis was applied to assess any undesirable beetroot soup characteristics. Color affects the attractiveness of food products, interfering with sensory evaluations and purchase decisions [33], particularly in beetroot formulations, where the characteristic red color is an indicator of pigment concentration and may comprise the most important attribute for product acceptance. The predominance of a red color was higher in pure beetroot soup when compared to microencapsulated beetroot soups in maltodextrin or starch. Although a red color was less present in starch beetroot soup, greater luminosity was observed.

The first property that consumers observe in every food product is appearance. Color can be altered during food processing due to pigment degradation, darkening reactions and/or compound oxidation. Some synthetic dyes, although largely applied in the food industry, can be harmful if consumed in high concentrations, while natural dyes have been proven to display anticancer and antioxidant properties [67]. Betalains, the set of aromatic indole pigments containing nitrogen and soluble in water, as already mentioned, confer color to beetroot and are categorized into two subclasses: betaxanthins, vulgaxanthin I, II and indicaxanthin, yellow pigments, and betacyanin, red pigments, mainly represented by betanin, (75–95%), the only red color pigment approved by the FDA to substitute synthetic pigments in the food industry [1,9]. The therapeutic properties of betanins, such as the prevention of neuronal, metabolic and cardiovascular diseases and cancer, are already recognized [9,14].

All these pigments seem to be preserved following beetroot soup microencapsulation and storage for at least 90 days, as the encapsulating agents act as physical obstacles that can reduce the effects of oxygen, light, heat and humidity on the encapsulated material, certainly contributing to consumer acceptance and purchase intention, as well as towards microencapsulated beetroot soup shelf life, as demonstrated previously [67].

Strict standards regarding the levels of these anions have been regulated in food and drinking water in the past. Until a decade ago, NO_3_^−^ was considered a toxic compound derived from unfavorable diets, as it was erroneously associated with the development of certain malignancies such as gastric cancer [8,14,15]. Therefore, in 1962, the World Health Organization (WHO) defined the maximum permissible limit of 3.7 mg of NO_3_^−^·kg^−1^ of body weight daily intake, the same content adopted by the European Food Safety Authority [14,15]. For a healthy 80 kg adult, this is equivalent to ~300 mg NO_3_^−^·day^−1^. The adoption of vegetarian diets, in general, however, increases NO_3_^−^ consumption by over 400 mg NO_3_^−^·day^−1^ for 80 kg adults, well above the acceptable daily intake values [8,15]. Nevertheless, studies carried out in this same line of research have failed to demonstrate a link between N-nitrosamines and NO_3_^−^ and NO_2_^−^ intake and cancer development in humans [8,14,15,20], making the cancer risk statement not true. Furthermore, interest in the biological function of this anion has increased due to the discovery of NO generation through the reduction of NO_3_^−^ to NO_2_^−^ and subsequently to NO, comprising an alternative route to the L-arginine/NO pathway [1,14].

When ingested, dietary NO_3_^−^ is absorbed in the proximal portion of the small intestine, where it accumulates endogenously as NO_3_^−^. About 30% of the NO_3_^−^ absorbed is extracted by the salivary glands, concentrated in saliva and reduced to NO_2_^−^ through nitrate-reductase [1,11]. Upon reaching the gastric acid medium, NO_2_^−^ is protonated, forming nitrous acid (HNO_2_), which spontaneously decomposes to NO and other bioactive nitrogen oxides. In addition, a small part of plasmatic NO_3_^−^ and NO_2_^−^ may be reduced to NO by xanthine oxidoreductase (XOR), deoxyhemoglobin (deoxyHb) and deoxymyoglobin (deoxyMb), aldehyde oxidase (AO), aldehyde dehydrogenase (ALDH) and carbonic anhydrase (CA) [11,20]. The produced NO crosses the endothelium and diffuses rapidly into smooth blood vessel muscle cells, activating soluble guanylate cyclase that converts GTP to cyclic GMP, decreasing Ca^2+^ cytosol concentrations. The low intracellular Ca^2+^ concentrations decrease complexation to calmodulin, promoting vascular smooth muscle cell relaxation, decreasing arterial stiffness and improving endothelial functional adaptation [15].

In the Western diet, about 85% of NO_3_^−^ intake comes from vegetables belonging to several botanica families [68]. Red beets contain 1300 mg of NO_3_^−^·kg^−1^ and are commonly used in dietary interventions to improve cardiovascular performance through decreased central blood pressure and arterial stiffness reduction, improving overall vascular function [6,13,16,69]. To promote metabolic NO production and improve hemodynamic and vascular parameters, i.e., reversing endothelial dysfunction and arterial stiffness in individuals presenting cardiovascular risk factors, dietary NO_3_^−^ supplementation should be long term (over 20 days) and greater than 370 mg (6.0 mmol) per day [2,11,12,16,69,70]. The high amounts of this vegetable to be ingested to achieve effective dietary NO_3_^−^ concentrations in the human body may be a limiting factor in ensuring adherence to long-term nutritional interventions [15,71]. The beetroot soups encapsulated at 1:2 and 1:1 (*w*/*w*) ratios, beet powder:encapsulating polysaccharide, contain NO_3_^−^ concentrations over 10 mmol·100 g^−1^. Thus, the novel microencapsulated beetroot soup technology was able to overcome the challenge, developing a high-acceptance beetroot soup containing pharmacological NO_3_^−^ concentrations in an adequate serving portion, presenting a long shelf life, easily reconstituted and consumed as a meal, favoring continuous intake and better adherence to a non-drug long-term-strategy therapy.

Saponins, triterpene glycosides in which the aglycone is covalently linked to one or two sugar chains through a glycosidic ester (C-28) or ether (C-3) bond [72], are widely distributed in edible vegetables, and exhibit antiviral, antidiabetic and antihemolytic properties [1,72]. In *Beta vulgaris* L., saponin concentrations can vary from 7.66 to 12.2 mg∙g^−1^ dry weight, but this vegetable may be enriched according to the final product processing [73]. Saponin contents are, for example, almost 3-fold concentrated in beetroot gel when compared to juice, ranging from 22 to 8.22 mg∙g^−1^, respectively [6]. If the saponin results of the present study are converted to mg∙g^−1^, pure and microencapsulated beetroot soup (at all ratios) contained three-fold higher saponin contents or more when compared to beetroot juice and beetroot gel.

Red beetroot is a tuber rich in several minerals, such as K (325 mg∙100 g^−1^), Mg (23 mg∙100 g^−1^), Mn (0.329 mg∙100 g^−1^), Na (78 mg∙100 g^−1^), P (40 mg∙100 g^−1^) and Zn (0.35 mg∙100 g^−1^) [74,75,76]. Higher mineral contents were noted for the powdered beetroot soups developed herein compared to in natura beetroot, except for Mn (0.237 mg∙100 g^−1^). Fresh beetroot contains 87.6 mg∙100 g^−1^ of water and nutrients and minerals are diluted in the aqueous medium in the vegetable matrix. Removing water by sublimation leads to an increased matrix density [75], increasing mineral concentrations in the powdered beetroot soups. 

The microencapsulated beetroot soups contain significantly lower mineral content when compared to pure beetroot soup formulations, as the food matrix was mixed at different ratios of the encapsulating polysaccharides in the microencapsulated beetroot soup formulations, resulting in lower mineral content. However, the beetroot soup microencapsulated in starch at the 1:1 and 1:2 (*w*/*w*) ratios presented mineral concentrations greater than or equal to raw beetroot. Mineral content variations seem to be associated to the type of microencapsulating agent and its behavior during lyophilization, as the capacity to hold in the core of the microparticles depends on the polysaccharide interaction with micronutrients [77]. Starch displayed a better performance as the microencapsulating agent for beetroot soup in the present study, considering all parameters.

As the aim of novel technological beetroot product development is to perform nutritional interventions in individuals at cardiovascular risk for CVD, overall product characteristics were carefully considered. The formulation of microencapsulated beetroot soups with starch and maltodextrin at a 1:2 (*w*/*w*) ratio chosen to provide dietary NO_3_^−^ included the polysaccharides as the encapsulating agents, which increased total dietary fiber and carbohydrate contents and, consequently, the energy value of the final product. The total dietary fibers in beetroot soup microencapsulated in starch and maltodextrin at a 1:2 (*w*/*w*) ratio were 3.57 ± 0.37 and 3.40 ± 0.36 g∙100 g^−1^, respectively, classifying microencapsulated beetroot soups as dietary fiber food sources, as they contain above 3 g∙100 g^−1^ of dietary fibers [78]. The consumption of fiber in food can lower bloodstream cholesterol levels, increasing the production of short-chain fatty acids and inactivating pathogenic bacteria by favoring the establishment of beneficial intestinal flora, which in turn boosts the immune system and prevents gastrointestinal infections [79].

Despite the use of olive oil in beetroot soup preparation, lipids comprised the macronutrients that contributed less to the energy value of the soups, which enables their classification as a low-fat food, in accordance with the current legislation for solid foods in Brazil [78]. Maintaining low lipid concentrations did not compromise beetroot soup flavor, texture or other organoleptic characteristics.

Betalains are easily degraded if exposed to light or oxygen, high water activity and physicochemical conditions or enzymes found in the gastrointestinal tract, reducing their absorption following oral administration and also leading to low bioavailability [80,81,82,83]. The microencapsulation of beetroot soups may favor the stability, bioaccessibility and bioavailability of betalains and other functional compounds. The encapsulation efficiency depends on the capacity of the employed polysaccharides to hold betalains within their microparticles, which will probably take place through hydrogen bonding. In the present study, both starch and maltodextrin trapped more than 50% of betalains in microcapsules, and encapsulation by lyophilization reduced water activity on betalains [84]. Betalains from cactus fruit and red dragon fruit have been microencapsulated in polysaccharides such as Arabic gum, pectin, xanthan gum and inulin, but few reports have evaluated encapsulation efficiency according to the encapsulating agent [77,85,86,87]. The microencapsulation efficiency of total betalains, betacyanins and betaxanthins in maltodextrin has been reported at low levels of 4%, 4.5% and 3.5%, respectively. In the same study, encapsulation with inulin retained only 2.6%, 2.8% and 2.3% of each pigment, respectively [77]. On the other hand, Vergara et al. [88] reported betacyanin and betaxanthin microencapsulation efficiencies of over 90% from purple cactus pear fruits in modified starch (Capsul^®^) by spray drying.

Although partial betalain microencapsulation was observed in pure beetroot soup in the present study, betalain content in the microcapsules was higher than doses considered as effective following pre-clinical and clinical assays [81,89,90,91]. In addition, betalain in pure beetroot soup may be prone to degradation, due to the harsher physicochemical conditions found in the gastrointestinal tract compared to non-encapsulated soup.

During the manufacturing and storage of the powdered beetroot soups, all procedures and analyses were performed according to the IN 60/2019, in addition to the RDC 331/2019 norm that establishes the microbiological analyses for ready-to-eat foods by the current Brazilian legislation [42,92]. The microbiological analysis findings demonstrated that the powdered beetroot soups comply with Brazilian and international regulatory agencies [42,93], indicating adequate microbiological quality for consumption, even after 30 days of storage. After 60 days, mold and yeast were observed only in the pure beetroot soup, and after 90 days in all powdered soup products. It is noteworthy that no safety limits for mold and yeast have been established, as the occurrence of pathogenic species in powdered food has not been reported and is not considered as contamination or as indicative of manufacturing safety failure. In general, the microbiological analysis findings during the product’s suggested shelf life indicate satisfactory hygienic processing conditions. However, the microencapsulated beetroot soups should be packed under vacuum and the finished product should be maintained under dry conditions and at room temperature.

The development of new products must be followed by acceptability assessments. Sensory analyses are an important tool in this process, comprising an interdisciplinary science that uses the complex interaction of the sense organs and the food’s chemical and physical characteristics to evoke, measure, evaluate and interpret sensorial characteristics and acceptability and/or preference for food products and many other materials. Sensory methods are based on responses to stimuli, which produce intensity, extension, duration, quality and pleasure or displeasure sensations [94]. In the present study, only powdered beetroot soup samples microencapsulated in starch and maltodextrin were evaluated in terms of sensory attributes, both presenting good scores regarding all attributes evaluated in the acceptance test (mainly color, texture and overall acceptability) and purchase intention. In contrast, the pure beetroot soup presented the lowest score (below 7.0) and purchase intention (below 4.0) compared to the microencapsulated beetroot soups for all evaluated attributes. This was maintained throughout the 90 evaluated days, with no variations over the evaluated periods. On the other hand, pure beetroot soup attributes evaluated at 60 and 90 days presented slight score variations, with slightly reduced scores. However, the flavor of the powdered products or the reconstituted soup was not assessed, as the aim was to carry out a predictive sensory analysis of the products following production within a predetermined storage period (90 days), i.e., sensory characteristics of the ready-to-be-marketed product (powder).

Studies on the psychological or nutritional factors that encourage consumers to accept or not a certain product are common, and some initially perceived attributes directly contribute to individual initial choices. In this regard, food appearance is the first attribute appreciated by humans, followed by odor, consistency, texture and flavor. Vision allows for assessments on color, shape, brightness and a multitude of attributes, leading to final positive or negative judgments. Consumers expect certain colors, as they commonly associate color to other characteristics, such as flavor and sweetness for example, so reaching acceptable food colors is paramount [95]. The starch- or maltodextrin-microencapsulated beetroot soups obtained excellent appearance results, even though they displayed different colors, which was in fact what was most noteworthy for the microencapsulated formulations.

The acceptability index of the beetroot soups reached 70%, allowing the products to be launched on the market [41]. All attributes received scores equal to or greater than 70%, suggesting that the use of encapsulating agents did not alter microencapsulated beetroot soup properties and taste acceptance. In addition, industrialized soups usually contain high amounts of sugar, chemical preservatives and dyes, but small amounts of dietary fiber. The significant challenge in the beetroot soup development was, therefore, to combine taste, appearance and nutritional quality. In this sense, the sensory evaluation demonstrated that the developed beetroot soup formulations present the sensorial characteristics expected for a well-designed food product, considering nutritional characteristics, antioxidant properties, NO_3_^−^ concentrations, dietary fiber contents and overall acceptability.

Encapsulation can improve the overall finished product, extending product stability and, consequently, shelf life, while still maintaining similar nutritional values and aromas between manufacturing and consumption times. Although encapsulation can be used as a means of masking the aroma or color of certain ingredients, which is of paramount importance in foods where flavor, aroma and visual appearance are usually key components affecting purchase decisions, the beetroot soups microencapsulated in starch or maltodextrin displayed overall acceptability, indicating a well-designed food product considering nutritional characteristics such as NO_3_^−^ and dietary fiber contents. The adherence to long-term supplementation with microencapsulated beetroot soups should, therefore, be successful following sensory valuation. Thus, the chronic consumption of microencapsulated beetroot soups may be achieved and applied to improve endothelial function with consequent blood pressure reduction in normal and hypertensive individuals, particularly those at high risk for cardiovascular diseases, also improving cerebrovascular hemodynamics.

## 5. Conclusions

Microencapsulation in starch or maltodextrin was successfully achieved for the development of a technological, ready-to-eat and tasty beetroot soup in order to formulate a food product especially designed to fulfill therapeutic purposes. Dietary NO_3_^−^, betacyanins and other antioxidant compounds present at effective concentrations in a powdered food product with extended shelf life are adequate for adherence to long-term supplementation for specific health purposes. The best microparticle performance considering morphology, size distribution, physicochemical and rheological features, shelf-life stability and sensory analyses was obtained by using starch as the encapsulating agent at a 1:2 (*w*/*w*) ratio. Therefore, the beetroot soup microencapsulated in starch can be employed as an adjuvant with vascular-protective effects in healthy, physically active individuals and in individuals who exhibit at least one CVD-related risk or those with established CVD. The findings reported herein encourage future studies concerning dietary NO_3_^−^ interventions with microencapsulated beetroot soup, including careful endothelial function and hemodynamic parameter evaluations in randomized controlled crossover trials.

## Figures and Tables

**Table 1:** Particle size distribution of beetroot soup microencapsulated by lyophilization.

Diameter Tests (μm)							
Beetroot Soup (g)	Starch (g)	Maltodextrin (g)	Encapsulating Matrix/Nucleus Ratio (*w*/*w*)	*d* (0.1)	*d* (0.5)	*d* (0.9)	*Span*
25	25	-	1:1	8.57 ± 1.83 ^e^	28.19 ± 2.19 ^e^	245.66 ± 2.31 ^d^	6.21 ± 0.31 ^a^
25	50	-	1:2	8.47 ± 1.29 ^e^	20.88 ± 2.92 ^f^	58.64 ± 2.22 ^f^	2.41 ± 0.22 ^c^
25	75	-	1:3	7.94 ± 1.74 ^e^	25.52 ± 2.55 ^e^	128.66 ± 2.14 ^e^	4.73 ± 0.48 ^b^
25	-	25	1:1	45.88 ± 2.08 ^a^	222.69 ± 2.39 ^a^	636.34 ± 2.04 ^a^	2.65 ± 0.86 ^c^
25	-	50	1:2	38.55 ± 2.44 ^b^	181.62 ± 2.11 ^b^	453.94 ± 2.03 ^b^	2.28 ± 0.49 ^c^
25	-	75	1:3	30.56 ± 1.66 ^c^	160.99 ± 2.75 ^c^	365.66 ± 2.01 ^c^	2.08 ± 0.81 ^c^
25	-	-	1:0	21.59 ± 2.11 ^d^	150.12 ± 2.66 ^d^	369.93 ± 2.16 ^c^	2.32 ± 0.61 ^c^

Values are expressed as means ± SD (n = 3). Different letters in the same column indicate differences between samples (*p* < 0.05). Results are displayed as d (0.1), d (0.5) and d (0.9), corresponding to the maximum size in µm of 10%, 50% and 90% of the analyzed particles. Beetroot soup at 1:0 with an encapsulating matrix/nucleus ratio of 1:0 does not contain encapsulating matrix (pure beetroot soup).

**Table 2:** Experimental design, yield, moisture, water activity (aw), water solubility and zeta potential (ZP) of microencapsulated beetroot soups following lyophilization.

Beetroot Soup (g)	Starch (g)	Maltodextrin (g)	Total Solids (%)	Beetroot Soup/Encapsulating Agent Ratio (*w*/*w*)	Yield (g/%)	Moisture (%)	*a_w_*	Solubility Index	Water Absorption Index	ZP
25	25	-	50	1:1	38.14 ± 1.40 g/77.68% ^e^	3.11 ± 0.63 ^b^	0.05 ± 0.01^c^	34.88 ± 1.55 ^b^	220.53 ± 1.79 ^b^	−14.51 ± 1.14 ^d^
25	50	-	75	1:2	65.87 ± 1.62 g/88.91% ^a^	3.15 ± 0.54 ^b^	0.07 ± 0.03 ^c^	33.18 ± 1.31 ^b^	221.98 ± 1.37 ^b^	−20.28 ± 1.04 ^a,b^
25	75	-	100	1:3	80.62 ± 1.51 g/82.13% ^b^	3.19 ± 0.55 ^b^	0.13 ± 0.07 ^c^	31.77 ± 2.39 ^b^	222.17 ± 1.85 ^b^	−21.29 ± 0.14 ^a^
25	-	25	50	1:1	38.33 ± 1.34 g/78.1% ^d^	4.61 ± 0.95 ^a,b^	0.41 ± 0.13 ^b^	52.35 ± 2.38 ^a^	508.04 ± 1.91 ^a^	−18.09 ± 1.06 ^b,c^
25	-	50	75	1:2	59.28 ± 1.82 g/80.25% ^c^	4.65 ± 0.84 ^a^	0.47 ± 0.11 ^b^	50.21 ± 1.88 ^a^	509.58 ± 1.88 ^a^	−19.16 ± 0.51 ^b,c^
25	-	75	100	1:3	74.41 ± 1.19 g/75.01% ^f^	4.77 ± 0.75 ^a^	0.69 ± 0.09 ^a^	48.29 ± 2.72 ^a^	509.83 ± 1.61 ^a^	−18.71 ± 0.15 ^c^
25	-	-	25	1:0	18.17 ± 0.90 g/73.6% ^g^	4.50 ± 0.71 ^a^	0.50 ± 0.15 ^a,b^	14.69 ± 1.83 ^c^	155.22 ± 1.57 ^c^	−8.36 ± 0.01 ^e^

Values are expressed as means ± SD. Different letters in the same column indicate statistically significant differences between samples (*p* < 0.05). *a_w_*, water activity; ZP, zeta potential. Beetroot soup with an encapsulating matrix/nucleus ratio of 1:0 does not contain encapsulating agent (pure beetroot soup).

**Table 3:** Color parameters, NO_3_^−^, NO_2_^−^ and average essential mineral contents of lyophilized beetroot soup formulations.

	Pure Beetroot Soup	Beetroot Soup in Starch Ratio (*w*/*w*)			Beetroot Soup in Maltodextrin Ratio (*w*/*w*)		
	1:0	1:1	1:2	1:3	1:1	1:2	1:3
Color parameters							
L*	10.11 ± 2.31	28.95 ± 3.72 ^c,*,#^	35.38 ± 2.23 ^b,*,#^	39.37 ± 1.13 ^a,*,#^	14.46 ± 2.15 ^c^	18.27 ± 1.71 ^b,#^	23.06 ± 1.44 ^a,#^
a*	9.88 ± 0.29	4.84 ± 1.07 ^a,*,#^	3.97 ± 0.41 ^a,*,#^	2.38 ± 0.34 ^b,*,#^	8.84 ± 0.67 ^a,#^	8.07 ± 0.32 ^a,#^	6.91 ± 0.49 ^b,#^
b*	0.66 ± 0.17	1.07 ± 0.15 ^a,#^	1.18 ± 0.18 ^a,#^	1.31 ± 0.22 ^a,#^	0.87 ± 0.45 ^a^	1.03 ± 0.22 ^a^	1.13 ± 0.26 ^a,#^
Bioactive compounds (100 g^−1^ fresh weight basis)							
NO_3_^−^ (mmol)	25.10 ± 0.08	16.67 ± 0.19 ^a,*,#^	10.46 ± 0.22 ^b,*,#^	4.55 ± 0.16 ^c,#^	15.98 ± 0.33 ^a,#^	8.43 ± 0.09 ^b,#^	4.23 ± 0.17 ^c,#^
NO_2_^−^ (mmol)	0.76 ± 0.11	0.39 ± 0.07 ^a,#^	0.14 ± 0.04 ^b,#^	0.07 ± 0.01 ^c,#^	0.34 ± 0.08 ^a,#^	0.17 ± 0.03 ^b,#^	0.05 ± 0.01 ^c,#^
Saponins (mg)	9922 ± 25.14	6544 ± 36.35 ^a,#^	3577 ± 45.60 ^b,#^	2071 ± 45.65 ^c,#^	6461 ± 69.72 ^a,#^	3480 ± 40.55 ^b,#^	2116 ± 40.35 ^c,#^
Minerals (mg·100 g^−1^ fresh weight basis)							
Na	477 ± 10.41	201 ± 9.56 ^a,*,#^	99.59 ± 13.23 ^b,*,#^	78.11 ± 5.11 ^c,*,#^	42.14 ± 7.39 ^a,#^	29.83 ± 5.31 ^b,#^	18.35 ± 2.53 ^b,#^
K	1220 ± 18.03	610 ± 6.62 ^a,*,#^	350 ± 7.64 ^b,*,#^	180 ± 5.06 ^c,*,#^	247 ± 8.51 ^a,#^	180 ± 10.13 ^b,#^	107 ± 11.23 ^c,#^
Mg	60.72 ± 5.18	32.45 ± 3.75 ^a,*,#^	19.76 ± 2.47 ^b,*,#^	10.27 ± 1.35 ^c,*,#^	10.01 ± 0.54 ^a,#^	7.03 ± 0.87 ^b,#^	4.86 ± 1.27 ^b,#^
Mn	0.24 ± 0.01	0.15 ± 0.05 ^a,*,#^	0.09 ± 0.01 ^b,*,#^	0.03 ± 0.03 ^c,*,#^	0.07 ± 0.01 ^a,#^	0.05 ± 0.01 ^b,#^	0.02 ± 0.03 ^c,#^
Zn	0.93 ± 0.04	0.28 ± 0.03 ^a,*,#^	0.15 ± 0.01 ^b,*,#^	0.07 ± 0.03 ^c,*,#^	0.07 ± 0.02 ^a,#^	0.03 ± 0.01 ^b,#^	0.02 ± 0.07 ^c,#^
P	20.64 ± 1.32	13.57 ± 0.98 ^a,*,#^	9.83 ± 0.08 ^b,*,#^	5.85 ± 0.04 ^c,*,#^	5.43 ± 2.29 ^a,#^	3.68 ± 1.19 ^a,#^	2.11 ± 1.06 ^a,#^
Total	1779 ± 7.18	858 ± 3.92 ^a,*,#^	493 ± 5.44 ^b,*,#^	274 ± 2.49 ^c,*,#^	304 ± 3.84 ^a,#^	221 ± 4.05 ^b,#^	132 ± 4.28 ^c,#^

Values are expressed as mean ± SD (n = 3). Different letters within the same line indicate differences between sample ratios ranging from 1:1, 1:2 and 1:3 ratios (*w*/*w*) at a significance level of *p* < 0.05. Asterisks denote differences from beetroot soup in maltodextrin at a significance level of *p* < 0.001. The symbol ^#^ denotes differences from pure beetroot soup at a significance level of *p* < 0.01. Beetroot soup with an encapsulating matrix/nucleus ratio of 1:0 does not contain encapsulating matrix (pure beetroot soup).

**Table 4:** Betalain, betacyanin and betaxanthin contents and encapsulation efficiencies (%).

Formulation	Betacyanin (mg·g^−1^)	Betaxanthin (mg·g^−1^)	Total Betalain (mg·g^−1^)	Encapsulation Efficiency		
Pure beetroot soup	258.1 ± 0.45 ^a^	142.2 ± 2.63 ^a^	400.2 ± 2.17 ^a^	Betacyanin	Betaxanthin	Betalain
Beetroot soup in starch at 1:2 (*w*/*w*) ratio	139.8 ± 3.21 ^b^	79.9 ± 4.31 ^b^	219.7 ± 4.92 ^b^	54.7 ± 0.85% ^a^	57.8 ± 1.50% ^a^	55.11 ± 0.66% ^a^
Beetroot soup in maltodextrin at 1:2 (*w*/*w*) ratio	141.5 ± 4.73 ^b^	82.4 ± 3.57 ^b^	223.9 ± 4.21 ^b^	55.5 ± 0.93% ^a^	58.4 ± 1.37% ^a^	56.2 ± 0.64% ^a^

Values are expressed as means ± SD (n = 3). Different letters between the columns indicate statistically significant differences between the powdered beetroot soups (*p* < 0.05). Beetroot soups were encapsulated in starch or maltodextrin at a 1:2 (*w*/*w*) ratio. Beetroot soup with an encapsulating matrix/nucleus ratio of 1:0 does not contain encapsulating matrix (pure beetroot soup).

**Table 5:** Sensory analysis and purchase intent of powdered beetroot soups.

Sensory Attributes	Powdered Beetroot Soup		
	Pure Beetroot Soup	Beetroot Soup in Starch (Ratio 1:2 *w*/*w*)	Beetroot Soup in Maltodextrin (Ratio 1:2 *w*/*w*)
0 days			
Color	6.92 ± 0.25 ^b,*,#^	7.83 ± 0.33 ^a^	7.78 ± 0.22 ^a^
Aroma	6.51 ± 0.13 ^b*,#^	7.17 ± 0.18 ^a^	7.11 ± 0.14 ^a^
Texture	6.77 ± 0.86 ^a,*^	7.89 ± 0.72 ^a^	7.36 ± 0.50 ^a^
Overall acceptability	6.95 ± 0.72 ^a,*,#^	7.97 ± 0.53 ^a^	7.71 ± 0.46 ^a^
Purchase intention	3.91 ± 0.14 ^a,*,#^	4.35 ± 0.28 ^a^	4.20 ± 0.31 ^a^
30 days			
Color	6.77 ± 0.33 ^b,*^	7.87 ± 0.21 ^a^	7.88 ± 0.49 ^a^
Aroma	6.63 ± 0.15 ^b,*,#^	7.10 ± 0.11 ^a^	7.38 ± 0.27 ^a^
Texture	6.96 ± 0.42 ^a,*,#^	7.99 ± 0.64 ^a^	7.22 ± 0.44 ^a^
Overall acceptability	6.34 ± 0.13 ^b,*,#^	7.91 ± 0.72 ^a^	7.37 ± 0.27 ^a^
Purchase intention	4.01 ± 0.36 ^a,*,#^	4.19 ± 0.23 ^a^	4.17 ± 0.13 ^a^
60 days			
Color	6.11 ± 0.22 ^b^	7.37 ± 0.31 ^a^	7.22 ± 0.26 ^a^
Aroma	5.89 ± 0.17 ^b^	7.19 ± 0.52 ^a^	7.29 ± 0.76 ^a^
Texture	5.73 ± 0.14 ^b^	7.55 ± 0.88 ^a^	7.41 ± 0.44 ^a^
Overall acceptability	5.81 ± 0.15 ^b,#^	7.79 ± 0.61 ^a^	7.56 ± 0.23 ^a^
Purchase intention	3.21 ± 0.16 ^b^	4.31 ± 0.48 ^a^	4.19 ± 0.27 ^a^
90 days			
Color	5.80 ± 0.74 ^b^	7.31 ± 0.33 ^a^	7.14 ± 0.36 ^a^
Aroma	5.77 ± 0.46 ^b^	7.24 ± 0.39 ^a^	7.20 ± 0.33 ^a^
Texture	5.81 ± 0.13 ^b^	7.33 ± 0.42 ^a^	7.25 ± 0.31 ^a^
Overall acceptability	5.15 ± 0.41 ^b^	7.81 ± 0.31 ^a^	7.44 ± 0.28 ^a^
Purchase intention	3.24 ± 0.17 ^b^	4.15 ± 0.16 ^a^	4.05 ± 0.11 ^a^

Values are expressed as means ± SD (n = 3). Different letters within the same line denote significant differences (one-way ANOVA, Bonferroni’s post-test; *p* < 0.001). The symbol * denotes difference from 60 days at a significance level of *p* < 0.001. The symbol ^#^ denotes difference from 90 days at a significance level of *p* < 0.001. Acceptance attributes considering aroma, color, taste and overall acceptability were assessed by applying a structured 9-point hedonic scale ranging from 1 = disliked it extremely to 9 = liked it very much. Purchase intention was evaluated using a structured 5-point hedonic scale ranging from 1 = would certainly not buy it to 5 = would certainly buy it.

## Data Availability

Data that support the findings of these experiments are available upon request.

## Supplementary Materials

The following supporting information can be downloaded at: https://www.mdpi.com/article/10.3390/foods12071497/s1, Table S1: General beetroot soup formulation; Table S2: ICP-OES parameters; Table S3: Elemental content in the certified reference materials, expressed as mg·kg^−1^ dry weight. Recoveries (%) were calculated for each element separately; Table S4: Limits of detection (LOD) and limits of quantification (LOQ) for each element determined in beetroot soup samples; Table S5: Proximate composition of lyophilized beetroot soup formulations (100 g); Table S6: Microbiological analyses of powdered beetroot soup: pure soup, soup microencapsulated in starch or maltodextrin at the ratio 1:2 (*w*/*w*).

## References

[1] Baião D.D.S., da Silva D.V., Del Aguila E.M., Paschoalin V.M.F. (2017). Nutritional, Bioactive and Physicochemical Characteristics of Different Beetroot Formulations. Food Additives.

[2] Lara J., Ashor A., Oggioni C., Ahluwalia A., Mathers J.C., Siervo M. (2016). Effects of inorganic nitrate and beetroot supplementation on endothelial function: A systematic review and meta-analysis. Eur. J. Nutr..

[3] Freitas C.S., da Silva G.A., Perrone D., Vericimo M.A., Baião D.D.S., Pereira P.R., Paschoalin V.M.F., Del Aguila E.M. (2019). Recovery of Antimicrobials and Bioaccessible Isoflavones and Phenolics from Soybean (Glycine max) Meal by Aqueous Extraction. Molecules.

[4] Trindade L., da Silva D., Baião D., Paschoalin V. (2021). Increasing the Power of Polyphenols through Nanoencapsulation for Adjuvant Therapy against Cardiovascular Diseases. Molecules.

[5] Baião D.D.S., Conte-Junior C.A., Paschoalin V.M.F., Alvares T.S. (2016). Quantitative and Comparative Contents of Nitrate and Nitrite in Beta vulgaris L. by Reversed-Phase High-Performance Liquid Chromatography-Fluorescence. Food Anal. Methods.

[6] da Silva D.V.T., Silva F.D.O., Perrone D., Pierucci A.P.T.R., Conte-Junior C.A., Alvares T.D.S., Del Aguila E.M., Paschoalin V.M.F. (2016). Physicochemical, nutritional, and sensory analyses of a nitrate-enriched beetroot gel and its effects on plasmatic nitric oxide and blood pressure. Food Nutr. Res..

[7] De Mejia E.G., Zhang Q., Penta K., Eroğlu A., Lila M.A. (2020). The colors of health: Chemistry, bioactivity, and market demand for colorful foods and natural food sources of colorants. Annu. Rev. Nutr..

[8] Chazelas E., Pierre F., Druesne-Pecollo N., Esseddik Y., de Edelenyi F.S., Agaesse C., De Sa A., Lutchia R., Gigandet S., Srour B. (2022). Nitrites and nitrates from food additives and natural sources and cancer risk: Results from the NutriNet-Santé cohort. Leuk. Res..

[9] da Silva D.V.T., Baião D.D.S., Ferreira V.F., Paschoalin V.M.F. (2020). Betanin as a multipath oxidative stress and inflammation modulator: A beetroot pigment with protective effects on cardiovascular disease pathogenesis. Crit. Rev. Food Sci. Nutr..

[10] Almeida C.C., Baião D.D.S., Rodrigues P.D.A., Saint’Pierre T.D., Hauser-Davis R.A., Leandro K.C., Paschoalin V.M.F., da Costa M.P., Conte-Junior C.A. (2022). Macrominerals and Trace Minerals in Commercial Infant Formulas Marketed in Brazil: Compliance with Established Minimum and Maximum Requirements, Label Statements, and Estimated Daily Intake. Front. Nutr..

[11] Lidder S., Webb A.J. (2013). Vascular Effects of Dietary Nitrate (as Found in Green Leafy Vegetables and Beetroot) via the Nitrate-Nitrite-Nitric Oxide Pathway. Br. J. Clin. Pharmacol..

[12] Baião D.D.S., Conte-Junior C.A., Paschoalin V.M.F., Alvares T.S. (2016). Beetroot juice increase nitric oxide metabolites in both men and women regardless of body mass. Int. J. Food Sci. Nutr..

[13] Baião D.D.S., D’El-Rei J., Alves G., Neves M.F., Perrone D., Del Aguila E.M., Paschoalin V.M.F. (2019). Chronic effects of nitrate supplementation with a newly designed beetroot formulation on biochemical and hemodynamic parameters of individuals presenting risk factors for cardiovascular diseases: A pilot study. J. Funct. Foods.

[14] Baião D.D.S., da Silva V., Paschoalin M. (2020). Beetroot, a Remarkable Vegetable: Its Nitrate and Phytochemical Contents Can be Adjusted in Novel Formulations to Benefit Health and Support Cardiovascular Disease Therapies. Antioxidants.

[15] Baião D.D.S., da Silva D.V.T., Paschoalin V.M.F. (2021). A Narrative Review on Dietary Strategies to Provide Nitric Oxide as a Non-Drug Cardiovascular Disease Therapy: Beetroot Formulations—A Smart Nutritional Intervention. Foods.

[16] Mattos S., Cunha M.R., Marques B.C., D´el-Rei J., Baião D.D.S., Paschoalin V.M.F., Oigman W., Neves M.F., Medeiros F. (2023). Acute effects of dietary nitrate on central pressure and endothelial function in hypertensive patients: A randomized, place-bo-controlled crossover study. Arq. Bras. De Cardiol..

[17] Baião D.D.S., De Freitas C.S., Gomes L.P., Da Silva D., Correa A.C.N.T.F., Pereira P.R., Del Aguila E.M., Paschoalin V.M.F. (2017). Polyphenols from Root, Tubercles and Grains Cropped in Brazil: Chemical and Nutritional Characterization and Their Effects on Human Health and Diseases. Nutrients.

[18] Baião D.D.S., Conte C.A., Silva J.T., Paschoalin V., Alvares T.S. (2013). L-Arginine Supplementation and Nitric Oxide Production:No Additional Effect When Associated to Exercise. Food Nutr. Sci..

[19] Woessner M.N., McIlvenna L., De Zevallos J.O., Neil C.J., Allen J.D. (2018). Dietary nitrate supplementation in cardiovascular health: An ergogenic aid or exercise therapeutic?. Am. J. Physiol. Circ. Physiol..

[20] McDonagh S.T.J., Wylie L., Thompson C., Vanhatalo A., Jones A.M. (2018). Potential benefits of dietary nitrate ingestion in healthy and clinical populations: A brief review. Eur. J. Sport Sci..

[21] Jesus R.P. Produção de Sopa Instantânea com Resíduos de Tambaqui (Colossoma Macropomum). Dissertação apresentada ao Programa de Pós-Graduação em Ciência de Alimentos da Universidade Federal do Amazonas (10 July 2015). https://tede.ufam.edu.br/handle/tede/4734.

[22] Gharsallaoui A., Roudaut G., Chambin O., Voilley A., Saurel R. (2007). Applications of spray-drying in microencapsulation of food ingredients: An overview. Food Res. Int..

[23] Silva I., Machado F., Moreno M., Nunes C., Coimbra M., Coreta-Gomes F. (2021). Polysaccharide Structures and Their Hypocholesterolemic Potential. Molecules.

[24] Lopez-Polo J., Silva-Weiss A., Giménez B., Cantero-Lopez P., Vega R., Osorio F.A. (2019). Effect of lyophilization on the physicochemical and rheological properties of food grade liposomes that encapsulate rutin. Food Res. Int..

[25] Prosapio V., Lopez-Quiroga E. (2020). Freeze-Drying Technology in Foods. Foods.

[26] Mazuco R.A., Cardoso P.M.M., Bindaco É.S., Scherer R., Castilho R.O., Faraco A.A.G., Ruas F.G., Oliveira J.P., Guimarães M.C.C., De Andrade T.U. (2018). Maltodextrin and Gum Arabic-Based Microencapsulation Methods for Anthocyanin Preservation in Juçara Palm (Euterpe edulis Martius) Fruit Pulp. Plant Foods Hum. Nutr..

[27] Maia P.D.D.S., dos Santos Baião D., da Silva V.P.F., Lemos Miguel M.A., Quirino Lacerda E.C., de Araújo Calado V.M., da Silva Carneiro C., Finotelli P.V., Pierucci A.P.T.R. (2020). Microencapsulation of a craft beer, nutritional composition, antioxidant stability, and drink acceptance. LWT.

[28] Maia P.D.D.S., Baião D.D.S., da Silva V.P.F., Calado V.M.D.A., Queiroz C., Pedrosa C., Valente-Mesquita V.L., Pierucci A.P.T.R. (2019). Highly Stable Microparticles of Cashew Apple (Anacardium occidentale L.) Juice with Maltodextrin and Chemically Modified Starch. Food Bioprocess Technol..

[29] Costa A., Nunes J., Lima B., Pedrosa C., Calado V., Torres A., Pierucci A. (2015). Effective stabilization of CLA by microencapsulation in pea protein. Food Chem..

[30] Association of Official Analytical Chemists (AOAC) (2012). Official Methods of Analysis.

[31] Favilla A.L.C., Junior E.R.d.S., Rodrigues M.C.N.L., Baião D.D.S., Paschoalin V.M.F., Miguel M.A.L., Carneiro C.D.S., Pierucci A.P.T.R. (2022). Microbial and physicochemical properties of spray dried kefir microcapsules during storage. LWT.

[32] Sosulski F.W. (1962). The centrifuge method for determining flour absorption in hard red spring wheats. Cereal Chem..

[33] Chandran J., Nisha P., Singhal R.S., Pandit A.B. (2014). Degradation of colour in beetroot (*Beta vulgaris* L.): A kinetics study. J. Food Sci. Technol..

[34] de Almeida C.C., Baião D.D.S., Rodrigues P.D.A., Saint’Pierre T.D., Hauser-Davis R.A., Leandro K.C., Paschoalin V.M.F., da Costa M.P., Conte-Junior C.A. (2022). Toxic Metals and Metalloids in Infant Formulas Marketed in Brazil, and Child Health Risks According to the Target Hazard Quotients and Target Cancer Risk. Int. J. Environ. Res. Public Health.

[35] Paschoalin V.M.F., Baião D.D.S., Silva F.D.O., D’El-Rei J., Neves M.F., Perrone D., Del Aguila E.M., Paschoalin V.M.F. (2018). A new functional beetroot formulation enhances adherence to nitrate supplementation and health outcomes in clinical practice. SDRP J. Food Sci. Technol..

[36] Tonon R.V., Brabet C., Hubinger M.D. (2009). Influência da temperatura do ar de secagem e da concentração de agente carreador sobre as propriedades físico-químicas do suco de açaí em pó. Food Sci. Technol..

[37] Kusznierewicz B., Mróz M., Koss-Mikołajczyk I., Namieśnik J. (2021). Comparative evaluation of different methods for determining phytochemicals and antioxidant activity in products containing betalains—Verification of beetroot samples. Food Chem..

[38] Stintzing F.C., Schieber A., Carle R. (2003). Evaluation of colour properties and chemical quality parameters of cactus juices. Eur. Food Res. Technol..

[39] McNamee B.F., O’Riorda E.D., O’Sullivan M. (2001). Effect of Partial Replacement of Gum Arabic with Carbohydrates on Its Microencapsulation Properties. J. Agric. Food Chem..

[40] American Public Health Association (APHA) American Water Works Association (AWWA) & Water Environment Federation (WEF). Standard Methods for the Examination of Water and Wastewater. https://www.mwa.co.th/download/file_upload/SMWW_1000-3000.pdf.

[41] Meilgaard M.C., Carr T., Civille G.V. (2015). Sensory Evaluation Techniques.

[42] Agência Nacional de Vigilância Sanitária (ANVISA) Padrões Microbiológicos de Alimentos e sua Aplicação (Resolução n° 331, de 23 de Dezembro de 2019). http://antigo.anvisa.gov.br/documents/10181/4660474/RDC_331_2019_COMP.pdf/c9282210-371f-4fb6-b343-7622ca9ec493.

[43] Rezende Y.R.R.S., Nogueira J.P., Narain N. (2018). Microencapsulation of extracts of bioactive compounds obtained from acerola (Malpighia emarginata DC) pulp and residue by spray and freeze drying: Chemical, morphological and chemometric characterization. Food Chem..

[44] Murali S., Kar A., Mohapatra D., Kalia P. (2015). Encapsulation of black carrot juice using spray and freeze drying. Food Sci. Technol. Int..

[45] Choi J., Wang S., Tung Y.-S., Morrison B., Konofagou E.E. (2010). Molecules of Various Pharmacologically-Relevant Sizes Can Cross the Ultrasound-Induced Blood-Brain Barrier Opening in vivo. Ultrasound Med. Biol..

[46] Bhandari B., Bansal N., Zhang M., Schuck P. (2013). Processes and Properties. Handbook of Food Powder Processes and Properties.

[47] Souza A.C.P. (2014). Caracterização e Estabilidade de Micropartículas de Antocianinas Extraídas do Bagaço da Produção do suco de Jabuticaba. Master’s Thesis.

[48] Maia P.D.D.S., Baião D.D.S., Nanini H.F., da Silva V.P.F., Frambach L.B., Cabral I.M., Pêgo B., Ribeiro B.E., Pavão M.S.G., Paschoalin V.M.F. (2022). Bioactive Compounds from *Pale Ale* Beer Powder Attenuate Experimental Colitis in BALB/c Mice. Molecules.

[49] Pashazadeh H., Zannou O., Ghellam M., Koca I., Galanakis C.M., Aldawoud T.M.S. (2021). Optimization and Encapsulation of Phenolic Compounds Extracted from Maize Waste by Freeze-Drying, Spray-Drying, and Microwave-Drying Using Maltodextrin. Foods.

[50] Devadiga D., Ahipa T. (2020). Betanin: A Red-Violet Pigment—Chemistry and Applications. Chem. Technol. Nat. Synth. Dye. Pigment..

[51] Sengupta D., Mondal B., Mukherjee K. (2015). Visible light absorption and photo-sensitizing properties of spinach leaves and beetroot extracted natural dyes. Spectrochim. Acta Part A Mol. Biomol. Spectrosc..

[52] Tan S.X., Ong H.C., Andriyana A., Lim S., Pang Y.L., Kusumo F., Ngoh G.C. (2022). Characterization and Parametric Study on Mechanical Properties Enhancement in Biodegradable Chitosan-Reinforced Starch-Based Bioplastic Film. Polymers.

[53] Abdullah A.H.D., Chalimah S., Primadona I., Hanantyo M.H.G. (2018). Physical and chemical properties of corn, cassava, and potato starchs. IOP Conf. Series: Earth Environ. Sci..

[54] Sritham E., Gunasekaran S. (2017). FTIR spectroscopic evaluation of sucrose-maltodextrin-sodium citrate bioglass. Food Hydrocoll..

[55] Muñoz D.P.V., Kurozawa L.E. (2020). Influence of combined hydrolyzed collagen and maltodextrin as carrier agents in spray drying of cocona pulp. Braz. J. Food Technol..

[56] Pena R.S., Mendonça N.B., Almeida M.D.C. (2010). Hygroscopic behavior of açaí powder. Braz. Mag. Agroind. Prod..

[57] Sun-Waterhouse D., Teoh A., Massarotto C., Wibisono R., Wadhwa S. (2010). Comparative analysis of fruit-based functional snack bars. Food Chem..

[58] Agência Nacional de Vigilância Sanitária (ANVISA) RDC n. 711, de 1° de Julho de 2022. Dispõe Sobre os Requisitos Sanitários dos Amidos, Biscoitos, Cereais Integrais, Cereais Processados, Farelos, Farinhas, Farinhas Integrais, Massas Alimentícias e pães. http://antigo.anvisa.gov.br/documents/10181/6482578/RDC_711_2022_.pdf/c739c4a9-6d94-424d-b27b-5ffed15474cf.

[59] Cano-Chauca M., Stringheta P., Ramos A., Cal-Vidal J. (2005). Effect of the carriers on the microstructure of mango powder obtained by spray drying and its functional characterization. Innov. Food Sci. Emerg. Technol..

[60] Costa S.J., Almeida A., Castro A., Domingues L., Besir H. (2013). The novel Fh8 and H fusion partners for soluble protein expression in Escherichia coli: A comparison with the traditional gene fusion technology. Appl. Microbiol. Biotechnol..

[61] Campelo-Felix P.H., Souza H.J.B., Figueiredo J.D.A., Fernandes R.V.D.B., Botrel D.A., de Oliveira C.R., Yoshida M.I., Borges S.V. (2017). Prebiotic Carbohydrates: Effect on Reconstitution, Storage, Release, and Antioxidant Properties of Lime Essential Oil Microparticles. J. Agric. Food Chem..

[62] Schäfer B., Hecht M., Harting J., Nirschl H. (2010). Agglomeration and filtration of colloidal suspensions with DVLO interactions in simulation and experiment. J. Colloid Interface Sci..

[63] Estevinho B.N., Damas A.M., Martins P., Rocha F. (2014). Microencapsulation of β-galactosidase with different biopolymers by a spray-drying process. Food Res. Int..

[64] Albert K., Tóth C., Verasztó B., Vatai G., Koris A. (2016). Microencapsulation Analysis Based on Membrane Technology: Basic Research of Spherical, Solid Precursor Microcapsule Production. Period. Polytech. Chem. Eng..

[65] Ahmad M., Mudgil P., Gani A., Hamed F., Masoodi F.A., Maqsood S. (2019). Nano-encapsulation of catechin in starch nanoparticles: Characterization, release behavior and bioactivity retention during simulated in-vitro digestion. Food Chem..

[66] dos Santos F.H., e Silveira B.M.P., de Souza L.L., Duarte A.K.C., Ribeiro M.C., Pereira K.C., da Costa J.M.G. (2020). Influence of wall materials on the microencapsulation of pequi oil by spray drying. Braz. J. Food Technol..

[67] Khazaei K.M., Jafari S., Ghorbani M., Kakhki A.H. (2014). Application of maltodextrin and gum Arabic in microencapsulation of saffron petal’s anthocyanins and evaluating their storage stability and color. Carbohydr. Polym..

[68] Blekkenhorst L.C., Bondonno N.P., Liu A.H., Ward N.C., Prince R.L., Lewis J.R., Devine A., Croft K.D., Hodgson J.M., Bondonno C.P. (2018). Nitrate, the oral microbiome, and cardiovascular health: A systematic literature review of human and animal studies. Am. J. Clin. Nutr..

[69] Kapil V., Khambata R., Robertson A., Caulfield M., Ahluwalia A. (2015). Dietary Nitrate Provides Sustained Blood Pressure Lowering in Hypertensive Patients: A randomized, phase 2, double-blind, placebo-controlled study. Hypertension.

[70] Bahadoran Z., Mirmiran P., Kabir A., Azizi F., Ghasemi A. (2017). The Nitrate-Independent Blood Pressure–Lowering Effect of Beetroot Juice: A Systematic Review and Meta-Analysis. Adv. Nutr. Int. Rev. J..

[71] Vasconcellos J., Silvestre D.H., Baião D.D.S., Werneck-De-Castro J.P., Alvares T.S., Paschoalin V.M.F. (2017). A Single Dose of Beetroot Gel Rich in Nitrate Does Not Improve Performance but Lowers Blood Glucose in Physically Active Individuals. J. Nutr. Metab..

[72] Chhikara N., Kushwaha K., Sharma P., Gat Y., Panghal A. (2019). Bioactive compounds of beetroot and utilization in food processing industry: A critical review. Food Chem..

[73] Mroczek A., Kapusta I., Janda B., Janiszowska W. (2012). Triterpene Saponin Content in the Roots of Red Beet (*Beta vulgaris* L.) Cultivars. J. Agric. Food Chem..

[74] Singh B., Hathan B.S. (2014). Chemical composition, functional properties and processing of beetroot—A review. Int. J. Sci. Eng. Res..

[75] U.S. Department of Agriculture (USDA) Beets, Raw. Vegetables and Vegetable Products, 2019. https://fdc.nal.usda.gov/fdc-app.html#/food-details/169145/nutrients.

[76] Mirmiran P., Houshialsadat Z., Gaeini Z., Bahadoran Z., Azizi F. (2020). Functional properties of beetroot (Beta vulgaris) in management of cardio-metabolic diseases. Nutr. Metab..

[77] Flores-Mancha M.A., Ruíz-Gutiérrez M.G., Sánchez-Vega R., Santellano-Estrada E., Chávez-Martínez A. (2020). Characterization of Betabel Extract (*Beta vulgaris*) Encapsulated with Maltodextrin and Inulin. Molecules.

[78] Agência Nacional de Vigilância Sanitária (ANVISA) Dispõe sobre o Regulamento Técnico sobre informação nutricional complementar. Ministério da Saúde. RDC n. 54, 12 de novembro de 2012. https://bvsms.saude.gov.br/bvs/saudelegis/anvisa/2012/rdc0054_12_11_2012.html.

[79] Jones J.M., Engleson J. (2010). Whole Grains: Benefits and Challenges. Annu. Rev. Food Sci. Technol..

[80] Frank T., Stintzing F.C., Carle R., Bitsch I., Quaas D., Straß G., Bitsch R., Netzel M. (2005). Urinary pharmacokinetics of betalains following consumption of red beet juice in healthy humans. Pharmacol. Res..

[81] Clifford T., Constantinou C.M., Keane K.M., West D.J., Howatson G., Stevenson E.J. (2017). The plasma bioavailability of nitrate and betanin from Beta vulgaris rubra in humans. Eur. J. Nutr..

[82] Zabot G.L., Rodrigues F.S., Ody L.P., Tres M.V., Herrera E., Palacin H., Córdova-Ramos J.S., Best I., Olivera-Montenegro L. (2022). Encapsulation of Bioactive Compounds for Food and Agricultural Applications. Polymers.

[83] Rocha F., Rezende J.D.P., Dias M.M.D.S., Pinto V.R.A., Stringheta P.C., Pires A.C.D.S., Vidigal M.C.T.R. (2023). Complexation of anthocyanins, betalains and carotenoids with biopolymers: An approach to complexation techniques and evaluation of binding parameters. Food Res. Int..

[84] Silva-Espinoza M.A., Ayed C., Foster T., Camacho M.D.M., Martínez-Navarrete N. (2019). The Impact of Freeze-Drying Conditions on the Physico-Chemical Properties and Bioactive Compounds of a Freeze-Dried Orange Puree. Foods.

[85] Otálora M.C., Carriazo J.G., Iturriaga L., Nazareno M.A., Osorio C. (2015). Microencapsulation of betalains obtained from cactus fruit (Opuntia ficus-indica) by spray drying using cactus cladode mucilage and maltodextrin as encapsulating agents. Food Chem..

[86] Li X., Zhang Z.-H., Qiao J., Qu W., Wang M.-S., Gao X., Zhang C., Brennan C.S., Qi X. (2022). Improvement of betalains stability extracted from red dragon fruit peel by ultrasound-assisted microencapsulation with maltodextrin. Ultrason. Sonochem..

[87] Janiszewska E. (2014). Microencapsulated beetroot juice as a potential source of betalain. Powder Technol..

[88] Vergara C., Saavedra J., Sáenz C., García P., Robert P. (2014). Microencapsulation of pulp and ultrafiltered cactus pear (Opuntia ficus-indica) extracts and betanin stability during storage. Food Chem..

[89] Tesoriere L., Butera D., Pintaudi A.M., Allegra M., A Livrea M. (2004). Supplementation with cactus pear (Opuntia ficus-indica) fruit decreases oxidative stress in healthy humans: A comparative study with vitamin C. Am. J. Clin. Nutr..

[90] Rahimi P., Mesbah-Namin S.A., Ostadrahimi A., Abedimanesh S., Separham A., Jafarabadi M.A. (2019). Effects of betalains on atherogenic risk factors in patients with atherosclerotic cardiovascular disease. Food Funct..

[91] da Silva D.V.T., Pereira A.D., Boaventura G.T., Ribeiro R.S.D.A., Verícimo M.A., de Carvalho-Pinto C.E., Baião D.D.S., Del Aguila E.M., Paschoalin V.M.F. (2019). Short-Term Betanin Intake Reduces Oxidative Stress in Wistar Rats. Nutrients.

[92] Agência Nacional de Vigilância Sanitária (ANVISA) Estabelece as Listas de Padrões Microbiológicos Para Alimentos (Instrução Normativa n° 60, de 23 de Dezembro de 2019). https://cvs.saude.sp.gov.br/zip/U_IN-MS-ANVISA-60_231219.pdf.

[93] World Health Organization (WHO) (1983). Microbiological Criteria for Foods: SUMMARY of Recommendations of FAO/OMS.

[94] Stone H., Sidel J.L. (2004). Sensory Evaluation Practices.

[95] Battistella N., Colombo J.R., Abreu K.C.K. (2010). A Importância da Cor nas Embalagens como Fator Influenciador no Momento da Compra.

