# Supplementary material for: Microencapsulated and Ready-to-Eat Beetroot Soup: A Stable and Attractive Formulation Enriched in Nitrate, Betalains and Minerals
Source: Foods. 2023 Apr 2;12(7):1497. doi: 10.3390/foods12071497 (PMC10093833; doi:10.3390/foods12071497)
Supplement: Supplementary file 1 [file foods-12-01497-s001.zip › foods-2272718-supplementary.pdf]

Supplementary files S1

Table S1. General beetroot soup formulation

Ingredient	Amount
Beetroot	1,764 g
Fresh garlic	22 g
Garlic powder	70 g
Onion powder	17g
Olive oil	15 mL
Parsley	13 g
Salt	10 g

Table S2. ICP-OES parameters

ICP-OES conditions	Values
Radiofrequency power (W)	1200
Plasma flow (L·min^{-1})	12
Auxiliary flow (L·min^{-1})	0.2
Nebulizer pressure (bar)	1.0
Sample flow rate (mL·min^{-1})	1.0
Integration time (s)	1.0
Resolution	High

Table S3. Elemental content in the certified reference materials, expressed as mg·kg^{-1} dry weight. Recoveries (%) were calculated for each element separately.

Reference materials (mg·kg^{-1})			
**Element**	**Reference**	**Experimental**	**Recovery**
Cu	4.9±0.2	5	102%
K	61000±2000	55000	91%
Mg	6500±300	6100	94%
Mn	21.2±1.0	22	104%
P	2600	2150	83%
Zn	15.6±1.2	13	83%

Supplementary files S4

Table S4. Limits of detection (LOD) and limits of quantification (LOQ) for each element determined in beetroot-soup samples

Minerals	LOD ($mg \cdot 100g^{-1}$)	LOQ ($mg \cdot 100g^{-1}$)
Cu	0.0051	0.0017
Na	0.031	0.099
K	0.014	0.046
Mg	0.020	0.066
Mn	0.0001	0.0002
P*	0.0025	0.0008
Zn	0.0002	0.0002

Table S5. Proximate composition of lyophilized beetroot soup formulations (100 g)

	Pure beetroot-soup	Beetroot-soup in starch (1:2)	Beetroot-soup in maltodextrin (1:2)
Ashes (%)	7.27±0.86[a]	2.22±0.44[b]	1.83±0.37[b]
Moisture (%)	4.50±0.34[a]	3.15±0.81[b]	4.65±0.53[a]
Energy (kcal)	353.01±2.35[b]	383.07±2.12[a]	384.64±1.77[a]
Carbohydrate (g)	71.70±0.97[b]	88.40±0.77[a]	89.20±0.79[a]
Protein (g)	3.92±0.26[a]	1.95±0.28[b]	1.91±0.13[b]
Lipid (g)	5.41±1.11[a]	2.27±0.82[b]	2.19±0.75[b]
Total dietary fibers (g)	6.35±0.22[a]	3.57±0.37[b]	3.40±0.36[b]
Total sugars (g)	21.31±1.24[a]	10.82±1.19[b]	12.55±1.39[b]
Fructose (g)	1.55±0.53[a]	0.68±0.55[b]	0.97±0.33[b]
Glucose (g)	3.14±0.61[a]	1.48±0.44[b]	1.98±0.28[b]
Sucrose (g)	16.62±1.15[a]	8.66±1.10[b]	9.60±1.07[b]

Values expressed as means ± standard deviation (n= 3). Different letters in the columns indicate statistically significant differences between samples ($p<0.05$).

Supplementary files S6

Table S6. Microbiological analyzes of powdered beetroot-soup: pure soup, soup microencapsulated in starch or maltodextrin at the ratio 1:2 (w/w)

**Microbiological analyses**	**Time (days)**												**Limits established by Brazilian current legislation**
	**0**			**30**			**60**			**90**			
	**PBS**	**BSA**	**BSM**	**PBS**	**BSA**	**BSM**	**PBS**	**BSA**	**BSM**	**PBS**	**BSA**	**BSM**	
Bacillus cereus **($CFU \cdot g^{-1}$)**	Absence	Absence	Absence	Absence	Absence	Absence	Absence	Absence	Absence	Absence	Absence	Absence	$3x10^3$ $CFU \cdot g^{-1}$
Salmonella sp. **(25 g)**	Absence	Absence	Absence	Absence	Absence	Absence	Absence	Absence	Absence	Absence	Absence	Absence	Absence
Esherichia coli **($MPN \cdot g^{-1}$)**	Absence	Absence	Absence	Absence	Absence	Absence	Absence	Absence	Absence	Absence	Absence	Absence	< 0.3 $MPN \cdot g^{-1}$
**Total coliforms ($MPN \cdot g^{-1}$)**	Absence	Absence	Absence	Absence	Absence	Absence	Absence	Absence	Absence	Absence	Absence	Absence	$1x10^2$ $MPN \cdot g^{-1}$
**Thermotolerant coliforms ($MPN \cdot g^{-1}$)**	Absence	Absence	Absence	Absence	Absence	Absence	Absence	Absence	Absence	Absence	Absence	Absence	$1x10^2$ $MPN \cdot g^{-1}$
**Molds and Yeasts ($CFU \cdot g^{-1}$)**	Absence	Absence	Absence	Absence	Absence	Absence	2.7×10^3	Absence	Absence	4.5×10^3	1.1×10^2	1.4×10^2	-

PBS. Pure beetroot soup; BSA, beetroot soup microencapsulated in starch; BSM, beetroot soup microencapsulated in maltodextrin; $CFU \cdot g^{-1}$, colony forming units per gram; $MPN \cdot g^{-1}$, most likely number per gram of powdered beetroot soup. Limits established by the current Brazilian legislation Resolution No. 331, of December 23, 2019[42].
